# Cystic fibrosis liver disease progression in the era of elexacaftor–tezacaftor–ivacaftor

**DOI:** 10.1016/j.jhepr.2025.101512

**Published:** 2025-07-05

**Authors:** Charlotte Mouliade, Lucia Parlati, Stylianos Tzedakis, Mathis Collier, Samir Bouam, Anais Vallet-Pichard, Valérie D’Halluin-Venier, Reem Kanaan, Stanislas Pol, Philippe Sogni, Pierre-Régis Burgel, Vincent Mallet, for the Demosthenes Research Group

**Affiliations:** 1 AP-HP Centre Université Paris Centre, Groupe Hospitalier Cochin Port Royal, DMU Cancérologie et Spécialités Médico-Chirurgicales, Service des Maladies du foie, Paris, France; 2 Université Paris Cité, F-75006, Paris, France; 3 AP-HP Centre, Groupe Hospitalier Cochin Port Royal, DMU Cancérologie et Spécialités Médico-Chirurgicales, Service de Chirurgie Digestive Hépatobiliaire et Endocrinienne, Paris, France; 4 AP-HP Centre, Groupe Hospitalier Cochin Port Royal, DMU Prime, Unité de Recherche Clinique, Paris, France; 5 AP-HP Centre Université de Paris, Groupe Hospitalier Cochin Port Royal, DMU PRIM, Service d’Information Médicale, Paris, France; 6 AP-HP Centre, Groupe Hospitalier Cochin Port Royal, DMU Thoros, Service de Pneumologie et Centre National de Référence de la Mucoviscidose, Paris, France

**Keywords:** Cystic fibrosis, Drug therapy, Chloride channel agonists, Therapeutic use, Liver diseases, Genetics, Mortality, Longitudinal study, Cohort study

## Abstract

**Background & Aims:**

The effect of elexacaftor–tezacaftor–ivacaftor (ETI) on cystic fibrosis liver disease (CFLD) outcomes remains unknown. Thus, we investigated the association between the ETI rollout and trends in CFLD progression in a nationwide cohort.

**Methods:**

Using the French National Hospital Discharge Database (2014–2023), we measured the incidence of CFLD progression (decompensated cirrhosis, gastroesophageal variceal bleeding, primary liver cancer, or liver transplantation) before and after ETI became available (December 2019) in patients with cystic fibrosis (pwCF) aged ≥12 years. Death without CFLD progression and lung transplantation were treated as competing events. Incidence rates were compared across ETI calendar eras in both the full cohort and a 1:1 propensity score-matched sample. Analyses used Kaplan–Meier curves, Fine–Gray competing risk models, and adjusted incidence rate ratios (aIRRs).

**Results:**

The cohort included 10,083 pwCF (median age: 19 years [IQR 14–29]; 52.6% male), with 24.6% censored pre-ETI and 75.4% post-ETI. The overall incidence of CFLD progression was 3.7 per 1,000 person-years: 25.4 pre-ETI versus 1.2 post-ETI (*p* <0.001). The incidence of all CFLD outcomes, including non-bleeding varices, declined post-ETI (*p* <0.001). In matched analyses, pwCF censored during the ETI era had a lower incidence of CFLD progression (aIRR 0.28, 95% CI: 0.18–0.43; *p* <0.001). In addition, deaths in pwCF occurred at an older age during the ETI era.

**Conclusions:**

The incidence of CFLD progression declined during the ETI rollout. While these findings suggest an association between ETI availability and improved liver outcomes, unmeasured confounders and concurrent changes in management might have also contributed. Thus, further studies are needed to confirm causality and understand underlying mechanisms.

**Impact and implications:**

In this nationwide French cohort study of over 10,000 people with cystic fibrosis, we observed a significant decline in the incidence of cystic fibrosis liver disease outcomes following the rollout of elexacaftor–tezacaftor–ivacaftor (ETI) in December 2019. The rate dropped from 25.4 to 1.2 per 1,000 person-years post-ETI. Matched analyses confirmed a reduced risk of liver disease progression, with an adjusted incidence rate ratio of 0.28. Although the findings suggest ETI may improve liver outcomes in people with cystic fibrosis, potential confounding factors necessitate further research to establish causality and understand the underlying biological mechanisms.

## Highlights

• Using nationwide French data, we evaluated liver disease progression among pwCF before and after ETI became available.
• Results showed 72% lower risk of severe liver complications after ETI introduction.
• Significant reductions in both overall mortality and the need for lung transplantation were observed following ETI rollout.
• ETI use was not associated with an increased risk of acute liver failure.
• These findings demonstrate a clear temporal association between ETI availability and improved liver outcomes in pwCF.

## Graphical abstract

**Figure:**
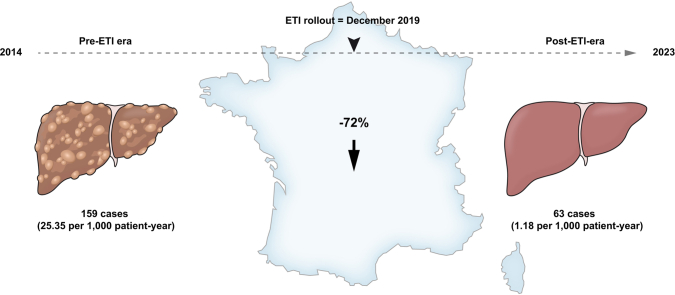

## Introduction

Cystic fibrosis is an autosomal recessive disorder caused by variants in the gene encoding cystic fibrosis transmembrane conductance regulator (CFTR), leading to multisystem involvement, particularly in the lungs, gastrointestinal system, pancreas, and biliary tract.^1^^,^^2^

Cystic fibrosis liver disease (CFLD) is a complication of cystic fibrosis, affecting an estimated 20–40% of patients with cystic fibrosis (pwCF). Among these, 5–10% progress to severe liver disease, characterized by portal hypertension and/or decompensated cirrhosis.[3], [4], [5], [6] Data from the Cystic Fibrosis Foundation Patient Registry (2003–2012) indicate 10-year cumulative rates of 6.6% for variceal bleeding, 9.9% for liver transplantation, and 6.9% for liver-related mortality,^7^ noting that some pwCF undergo liver transplantation without CFLD progression to a liver-related complication.

The advent of CFTR modulators (Table 1), particularly the triple combination therapy elexacaftor–tezacaftor–ivacaftor (ETI), has revolutionized the management of cystic fibrosis.^2^ ETI was initially developed in pwCF carrying at least one F508del variant, the most common CFTR variant worldwide, but it is also effective in pwCF carrying non-F508del variants, making this therapy suitable for up to 92% of pwCF.^8^

**Table 1:** Approved CFTR modulator treatments for pwCF.

Trade name	Active substance	ATC code	Filing date
Kalydeco	Ivacaftor	R07AX02	1 January 2012
Orkambi	Lumacaftor, ivacaftor	R07AX30	2 July 2015
Symkevi	Tezacaftor, ivacaftor	R07AX31	29 November 2018
Kaftrio∗	ETI∗	R07AX32∗	7 August 2020

Source: European Medicines Agency.

CFTR, cystic fibrosis transmembrane conductance regulator; ETI, elexacaftor–tezacaftor–ivacaftor; pwCF, patients with cystic fibrosis.

∗ In France, ETI was made available via a nominative Temporary Use Authorization in December 2019.

In France, ETI became available via an early-access program in December 2019. Over the subsequent 5 years, significant advances in managing pulmonary complications were documented.^2^ However, the impact of ETI therapy on CFLD progression remains uncertain. Advanced liver disease was an exclusion criterion in all clinical trials evaluating CFTR modulators,[9], [10], [11] leaving a critical gap in understanding its effectiveness on CFLD outcomes. Consequently, current guidelines recommend neither for nor against CFTR modulator use in pwCF with CFLD.^12^

To address this evidence gap, we conducted a nationwide cohort study of pwCF hospitalized in France between 2014 and 2023. In the absence of individual-level data on ETI exposure, we adopted a calendar-based exposure definition, leveraging the progressive rollout of ETI from December 2019 onward. Our objective was to compare the incidence of CFLD progression before and after the availability of ETI, and to generate population-level evidence for its potential hepatic benefit in pwCF.

## Methods

### Data source

Data were sourced from the French National Hospital Discharge Database (*Programme de Médicalisation des Systèmes d’Information; PMSI*). During the observational period, the entire French population (68.1 million in 2020) had equal access to universal, tax-financed healthcare services. The database contains anonymized, standardized discharge summaries that provide detailed patient demographics (age, sex, and postal code of residency), primary and associated discharge diagnoses coded using the WHO International Classification of Diseases, Tenth Revision (ICD-10), medical procedures, discharge dates, length of stay, and entry and discharge modes, including in-hospital mortality. Each patient’s historical and underlying conditions were traced back to 2014 using unique anonymous identifiers.^13^ The PMSI coding system has demonstrated 100% specificity for hard outcomes, including liver-related complications and in-hospital mortality. For sensitivity analyses and validation of our approach, see the study by Schwarzinger and colleagues.^14^ Missingness was minimal (2.3% of deprivation score) for primary outcomes and key covariates. No imputation was performed; complete-case analyses were used. The study was approved by the *Commission Nationale de l’Informatique et des Libertés* under registration number DR-2017-404. We used deidentified data, and informed consent was not required.

### Study population

We included all patients (ICD-10: E84%) aged 12 years or older with cystic fibrosis as a primary or associated discharge diagnosis between January 2014 and December 2023 (N = 12,625). To reduce the risk of false positivity, we retained pwCF with at least one primary discharge diagnosis of cystic fibrosis with pulmonary manifestations (E840, n = 9,833) or intestinal manifestations (E841, n = 1,165). pwCF who underwent liver transplantation without previous evidence of CFLD progression were excluded from the analysis.

### Exposures

Individual-level data on ETI treatment initiation, genotype eligibility, and adherence are not available in the PMSI. Consequently, exposure was defined based on censoring during the ETI era, which began in France in December 2019 with the launch of the early-access program for pwCF aged ≥12 years. In the absence of individual treatment data, this calendar-based proxy was adopted to approximate ETI exposure. According to national registry data, ETI uptake increased progressively, with ∼5.7% of eligible individuals treated in 2020 and 66.1% by 2023.^15^ These estimates are based on the entire cystic fibrosis population and included ∼900 lung-transplanted individuals and 400 children under the age of 2 years, who were not eligible for ETI. As a result, these uptake figures likely underestimate the true proportion of eligible patients who received ETI. Additional exposures included alcohol use disorders, smoking, chronic HBV or HCV, non-viral etiologies of chronic liver disease (*e.g.* congenital, autoimmune, or metabolic conditions), type 2 diabetes mellitus, obesity, major comorbidities, and socioeconomic deprivation. Definitions for alcohol use disorders, diabetes, and obesity are provided in the study by Mallet and colleagues.^16^ Frailty was assessed using the Charlson Comorbidity Index (CCI), categorized as low (<3), intermediate (3–5), or high (>5). Socioeconomic status was evaluated using the French Deprivation Index, stratified into quintiles.^17^^,^^18^ All covariates were coded as binary variables, except for age, which was modeled as a continuous variable. The complete codebook is provided in Table S1.

### Outcome measure

The primary outcome was CFLD progression, defined as the first occurrence of decompensated cirrhosis (ascites, hepatic encephalopathy, or non-obstructive jaundice in patients with cirrhosis, excluding those with primary liver cancer), primary liver cancer (including hepatocellular carcinoma and intrahepatic cholangiocarcinoma), or gastroesophageal variceal bleeding. Gastroesophageal variceal bleeding without cirrhosis was observed in 31 cases (16.2%). Non-bleeding gastroesophageal varices were defined as the record of gastroesophageal varices or band ligation without a previous record of gastroesophageal variceal bleeding. We chose to censor pwCF at the time of lung transplantation, because this intervention can alter the natural course of CFLD. The secondary outcome was mortality.

### Statistical analysis

Our objective was to compare the incidence rates and adjusted incidence rate ratios (aIRRs) of CFLD progression across the pre-ETI and ETI calendar periods. Given the gradual uptake of ETI and the absence of individual-level treatment exposure data, a causal inference framework was not applicable. Therefore, we adopted a calendar-based exposure definition, anchored in the timeline of national ETI rollout. To mitigate confounding, we performed 1:1 propensity score matching (PSM) by ETI period (caliper 0.1), including follow-up duration as a covariate to adjust for potential lead-time bias, under the assumption that longer follow-up might reflect better underlying health. Covariate balance post matching is shown in Table S2. Incidence rates (per 1,000 person-years) were estimated and compared using Poisson regression with the logarithm of follow-up time as an offset, in both the unmatched and PSM samples. aIRRs were derived from multivariable Poisson models. All analyses were conducted using R (version 4.4.0, *Puppy Cup*; R Foundation for Statistical Computing, Vienna, Austria), with a two-sided significance threshold of *p* <0.05.

## Results

### pwCF cohort characteristics

The cohort included 10,083 pwCF aged ≥12 years, with a median age at inception of 19 years (IQR: 14–29) and a balanced sex distribution (52.6% male). Smoking and alcohol use disorders were recorded in 6.3% and 3.0% of patients, respectively. Liver-specific risk factors were present in 1.7% of the cohort. Diabetes mellitus or obesity affected 27.1% of patients. Overall, 24.6% were censored during the pre-ETI era and 75.4% during the ETI era. Patients censored during the ETI era were older at the time of censoring (*p* <0.001) and had longer follow-up durations (*p* <0.001). Smoking (*p* <0.001) and obesity (*p* <0.001) were more frequent in the ETI group, whereas liver risk factors (*p* <0.001), diabetes mellitus (*p* <0.001), and social deprivation (*p* = 0.016) were more prevalent in the pre-ETI group. Sex (*p* >0.9), comorbidity burden (*p* = 0.7), alcohol use disorders (*p* = 0.2), and HIV infection (*p* = 0.2) did not differ significantly between groups. Additional characteristics are detailed in Table 2.

**Table 2:** Characteristics of pwCF aged 12 years and older across ETI eras.

**Characteristic**	**Overall** N = 10,083∗	**Censored during pre-ETI era** (n = 2,476, 24.6%)∗	**Censored during ETI era** (n = 7,607, 75.4%)∗	*p***value**†
Age at inception (years)	19 (14, 29)	18 (14, 26)	19 (12, 30)	0.083
Age at censoring (years)	24 (18, 36)	20 (18, 29)	26 (18, 38)	<0.001
Male sex	5,301 (52.57)	1,302 (52.58)	3,999 (52.57)	>0.9
Smoking habits	634 (6.29)	117 (4.73)	517 (6.80)	<0.001
Alcohol use disorders	308 (3.05)	65 (2.63)	243 (3.19)	0.2
Liver risk factors	167 (1.66)	67 (2.71)	100 (1.31)	<0.001
Chronic HBV	37 (0.37)	12 (0.48)	25 (0.33)	0.3
Chronic HCV	37 (0.37)	18 (0.73)	19 (0.25)	<0.001
Chronic HDV	4 (0.04)	2 (0.08)	2 (0.03)	0.3
Non-viral causes of chronic liver disease	106 (1.05)	46 (1.86)	60 (0.79)	<0.001
Diabetes mellitus	2,341 (23.22)	808 (32.63)	1,533 (20.15)	<0.001
Obesity	582 (5.77)	81 (3.27)	501 (6.59)	<0.001
HIV infection	39 (0.39)	6 (0.24)	33 (0.43)	0.2
CCI ≥3	1,416 (14.04)	341 (13.77)	1,075 (14.13)	0.7
Deprivation quintile 4 or 5	3,710 (37.69)	960 (39.75)	2,750 (37.02)	0.016
Follow-up duration (months)	78 (35, 110)	29 (12, 47)	97 (63, 114)	<0.001

This comparison captures temporal associations and does not reflect individual-level exposure to ETI. The ETI era started in France after December 2019. Patients were censored at the time of CFLD progression or upon undergoing lung transplantation without previous CFLD progression. The CCI was categorized into low (<3), intermediate (3–5), and high (>5) scores to reflect increasing levels of frailty. The French Deprivation Index was used to assess spatial socioeconomic and health inequalities, with scores divided into quintiles.

CCI, Charlson Comorbidity Index; ETI, elexacaftor–tezacaftor–ivacaftor; FDEP, French Deprivation Index.

∗ Data are presented as median (Q1, Q3) or *n* (%).

† Wilcoxon rank sum test; Pearson’s Chi-square test; Fisher’s exact test.

### Incidence of CFLD progression across ETI eras in the complete cohort

The cumulative incidence of CFLD progression (Fig. S1) was significantly lower among pwCF censored during the ETI era (*p* <0.001, Fine–Gray test). Overall, the incidence of CFLD progression was 3.7 per 1,000 person-years, decreasing from 25.4 during the pre-ETI era to 1.2 per 1,000 person-years during the post-ETI era (*p* <0.001). All liver-related events declined significantly during the ETI era (Table 3). The incidence of liver transplantation dropped from 4.7 to 0.04 per 1,000 person-years, whereas lung transplantation without previous CFLD progression decreased from 81.1 to 1.6 per 1,000 person-years (*p* <0.001 for both). Records of non-bleeding gastroesophageal varices and/or band ligations were also significantly less common during the post-ETI era (*p* <0.001). Acute liver failure did not increase during the post-ETI era (*p* <0.001). Overall mortality declined more than 15-fold (*p* <0.001). Despite the marked reduction in liver-related outcomes, the median age at CFLD progression did not differ significantly between eras, whereas the age at death was ∼10 years higher during the post-ETI era (*p* <0.001; Table S2).

**Table 3:** Incidence of CFLD progression in pwCF aged 12 years and older across ETI eras.

**Event**	**Overall**∗	**Pre-ETI incidence**∗	**Post-ETI incidence**∗	**Adjusted IRR (95% CI)**	*p***value**
CFLD progression	222 (3.72)	159 (25.35)	63 (1.18)	0.06 (0.05–0.08)	<0.001
Gastroesophageal varices (non-bleeding)	166 (2.78)	75 (11.96)	91 (1.7)	0.22 (0.15–0.3)	<0.001
Gastroesophageal varices (bleeding)	77 (1.29)	55 (8.77)	22 (0.41)	0.06 (0.04–0.11)	<0.001
Decompensated cirrhosis	172 (2.88)	126 (20.09)	46 (0.86)	0.06 (0.04–0.09)	<0.001
Hepatocellular carcinoma	12 (0.2)	9 (1.43)	3 (0.06)	0.03 (0.01–0.13)	<0.001
Acute liver failure	146 (2.45)	90 (14.35)	56 (1.05)	0.08 (0.06–0.12)	<0.001
Liver transplantation	28 (0.47)	26 (4.14)	2 (0.04)	0.02 (0–0.08)	<0.001
Lung transplantation	593 (9.94)	509 (81.14)	84 (1.57)	0.02 (0.02–0.03)	<0.001
Overall mortality	565 (9.47)	370 (58.98)	195 (3.65)	0.06 (0.05–0.07)	<0.001

The total follow-up time was 75,276 person-months (6,273 person-years) during the pre-ETI era and 640,659 person-months during the post-ETI era (i.e. 53,388 person-years). aIRRs and *p* values were estimated from Poisson regression models adjusted for age, sex, smoking, alcohol use disorders, liver and metabolic risk factors, major comorbidities, and social deprivation with an offset for log(person-time). This comparison captures temporal associations and does not reflect individual-level exposure to ETI.

aIRRs, adjusted incidence rate ratios; CFLD, cystic fibrosis liver disease; ETI, elexacaftor–tezacaftor–ivacaftor.

∗ Data are presented as *n* (%).

### Incidence of CFLD progression across ETI eras in matched patients

**Fig. 1:**
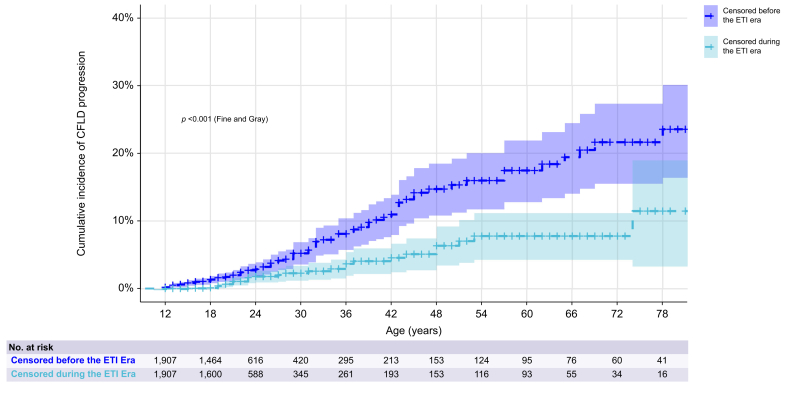
Cumulative incidence of CFLD progression in matched pwCF aged ≥12 years across ETI eras. Kaplan–Meier estimates of CFLD progression probability in pwCF aged 12 years and older, stratified by whether they were censored before or after the introduction of ETI on December 15, 2019. This comparison reflects temporal trends, but does not represent individual-level exposure to ETI. CFLD progression was any of decompensated cirrhosis, portal hypertension bleeding, primary liver cancer, liver transplantation, or lung transplantation without previous CFLD progression. CFLD, cystic fibrosis liver disease; ETI, elexacaftor–tezacaftor–ivacaftor; pwCF, patients with cystic fibrosis.

**Fig. 2:**
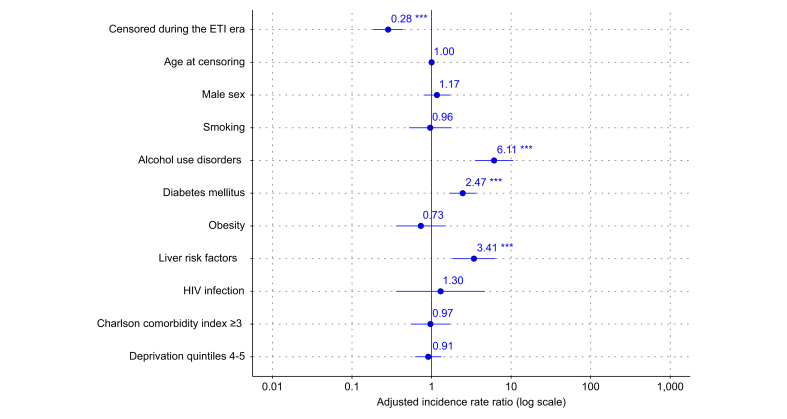
Adjusted incidence rate ratios for CFLD progression in matched pwCF aged ≥12 years across ETI eras. Adjusted incidence rate ratios were estimated from a weighted multivariable Poisson regression model assessing the association between censoring before or after the introduction of ETI (15 December 2019) and CFLD progression. Time at risk was included as a log-transformed offset term. Results are based on the propensity score-matched sample by ETI period (see Fig. S2 for covariate balance). CFLD, cystic fibrosis liver disease; ETI, elexacaftor–tezacaftor–ivacaftor; pwCF, patients with cystic fibrosis.

The cumulative incidence of CFLD progression in the PSM cohort by ETI period (n = 3,814; see Fig. S2 for covariate balance) was significantly lower among pwCF censored during the ETI era (*p* <0.001, Fine–Gray test; Fig. 1). Follow-up duration was not associated with the risk of CFLD progression in the matched cohort (*p* = 0.2). Other risks for CFLD progression are detailed in Table S3. In the corresponding multivariable analysis, censoring during the ETI era remained independently associated with a significantly lower incidence of CFLD progression, with an aIRR of 0.28 (95% CI: 0.18–0.43; *p* <0.001). Other independent risk factors included alcohol use disorders (*p* <0.001), diabetes mellitus (*p* <0.001), and liver-related comorbidities (*p* = 0.001) (Fig. 2).

## Discussion

In this nationwide analysis, we observed a marked reduction in the incidence of CFLD progression and all-cause in-hospital mortality among French pwCF aged ≥12 years during the period following the introduction of ETI. In a PSM cohort designed to reduce measured confounding, censoring during the ETI era was associated with a >70% lower incidence rate of CFLD progression (aIRR 0.28, 95% CI: 0.18–0.43; *p* <0.001). Notably, the incidence of acute liver failure did not increase during the post-ETI period.

This study addresses a key knowledge gap identified in recent guidelines for the management of hepatobiliary complications in pwCF.^12^ To our knowledge, it provides the first nationwide population-based analysis of CFLD outcomes across the ETI rollout period. The observed reduction in severe liver-related events during this timeframe suggests that ETI has a role in modifying the hepatic trajectory of pwCF. However, in the absence of individual treatment data, this association remains hypothesis generating.

Our findings align with small observational studies reporting improved liver parameters under CFTR modulator therapy.[19], [20], [21], [22], [23], [24] Although reports on liver stiffness changes remain inconsistent,^19^^,^^25^ reductions in liver transplantation rates in our study mirror trends reported in UNOS registry data,^26^ and parallel the decline in lung transplantation rates observed in the USA post-ETI rollout.^27^ Importantly, we found no evidence of increased risk of acute liver failure during the ETI era, consistent with pediatric safety reports.^28^

CFTR is expressed in the biliary epithelium, where dysfunction alters bile secretion and flow, contributing to cholangiopathy and eventually portal hypertension.^29^^,^^30^ ETI improves CFTR function and might thereby enhance bile fluidity and flow, potentially reducing biliary obstruction, inflammation, and fibrosis.^31^ Although the biliary phenotype of CFLD could plausibly respond to CFTR modulation, the vascular subtype, characterized by non-cirrhotic portal hypertension, has an uncertain pathogenesis and its response to ETI is unknown.

Although the observed decrease in portal hypertension-related complications during the ETI era is consistent with a plausible biological mechanism, this interpretation should be viewed with caution. Changes in clinical practice, supportive care, nutritional status, or hospitalization thresholds over time could also account for some of the observed trend. Furthermore, our reliance on calendar time as a proxy for ETI exposure limits any causal interpretation. The reduction in non-liver-related outcomes (*e.g.* lung transplantation and mortality) might reflect overall improvements in care, beyond the specific hepatic effects of ETI.

Our study has limitations. First, individual-level data on ETI initiation, duration, adherence, and genotype eligibility were not available, precluding any direct inference about drug efficacy. We also had no information on previous CFTR modulator therapies. Second, we relied on discharge diagnoses to identify outcomes, which can be subject to under- or over-coding. However, the specificity of PMSI coding for severe liver-related complications is high, and our outcome definitions were conservative. Third, we were unable to capture subclinical or early-stage liver disease, which might precede overt CFLD events. Fourth, although PSM was used to balance observed covariates, unmeasured confounding, such as nutritional status, adherence to supportive treatments, or evolving care standards, might have contributed to the findings. The gradual ETI uptake,^15^ together with differential survival, introduces the risk of immortal time bias, although we accounted for follow-up duration in all models. Finally, the use of a calendar-based exposure design, although necessitated by data limitations, restricted our ability to emulate a target trial and prevented the use of causal language.

In summary, we report a temporal association between the ETI era and a lower burden of CFLD progression and mortality in pwCF at the national level. Although these findings might reflect a hepatic benefit of ETI, they should be interpreted as preliminary and exploratory. The results do not establish causality, but do provide a strong rationale for further research. Future prospective studies should aim to combine clinical, biological, and imaging data to better characterize early hepatic changes under ETI and distinguish between subtypes of CFLD. Individual-level data on ETI exposure will be crucial to clarify treatment effects and inform personalized care. Until such data are available, our study offers important population-level insights that could inform clinical vigilance, guideline development, and research priorities in the management of CFLD during the ETI era.

## Abbreviations

aIRRs, adjusted incidence rate ratios; CCI, Charlson Comorbidity Index; CFLD cystic fibrosis liver disease; CFTR, cystic fibrosis transmembrane conductance regulator; ETI, ,elexacaftor–tezacaftor–ivacaftor; FDEP, French Deprivation Index; ICD-10, International Classification of Diseases, Tenth Revision; PMSI, Programme de Médicalisation des Systèmes d’Information; PSM, propensity score matching; pwCF, patients with cystic fibrosis.

## Financial support

The study did not receive any external funding.

## Authors’ contributions

Conception of the study, analysis and interpretation of the data, drafting of manuscript: VM. Analysis and interpretation of the data, editing of manuscript: CM, LP, ST, MC, PS, SB, RK, PRB. Acquisition of data, editing of manuscript: SB. Statistical analysis supervision: MC. Responsible for the content of the report: VM. All authors have seen and approved the final version of the manuscript.

## Data availability

None.

## Conflicts of interest

VM reports a role as a coinvestigator for Genfit, Intercept, Janssen, Novo-Nordisk, and Galmed; and travel fees from AbbVie. SP reports consulting and lecturing fees from Gilead, Abbvie, NovoNordisk, Pfizer, Viiv, and LFB, and grants from Gilead and Abbvie. PRB reports consulting fees from Astra Zeneca, Chiesi, Insmed, Pfizer, Sanofi, MSD, Viatris, and Vertex; and travel fees Astra Zeneca, Chiesi. The remaining authors have no conflicts of interest to report.

Please refer to the accompanying ICMJE disclosure forms for further details.
