# Supplementary material for: Cystic fibrosis liver disease progression in the era of elexacaftor–tezacaftor–ivacaftor
Source: JHEP Rep. 2025 Jul 5;7(10):101512. doi: 10.1016/j.jhepr.2025.101512 (PMC12506492; doi:10.1016/j.jhepr.2025.101512)
Supplement: Multimedia component 1 [file mmc1.pdf]

Cystic fibrosis liver disease progression in the era of elexacaftor–tezacaftor–ivacaftor

Charlotte Mouliade, Lucia Parlati, Stylianos Tzedakis, Mathis Collier, Samir Bouam, Anais Vallet-Pichard, Valérie D'Halluin-Venier, Reem Kanaan, Stanislas Pol, Philippe Sogni, Pierre-Régis Burgel, Vincent Mallet, for the Demosthenes research group

Table of contents

Other members of the Demosthenes Group 2

Table S1 3

Table S2 7

Table S3 8

Fig. S1 9

Fig. S2 10

Other members of the Demosthenes Group

Marion Corouge,[1] Daniel Karinthi, [1] for the Demosthenes research group

1. AP-HP.Centre Université Paris Centre, Groupe Hospitalier Cochin Port Royal, DMU Cancérologie et spécialités médico-chirurgicales, Service des Maladies du Foie, Paris, France

**Table S1: Code Dictionary**	
Diagnosis	ICD-10/medical procedure codes
**Acute and Subacute Hepatic Failure**	K72.0, K71.2
**Acute Hepatitis a with Hepatic Coma**	B15.0
**Acute Hepatitis a without Hepatic Coma**	B15.9
**Acute Hepatitis B with Delta Agent**	B16.0, B16.1, B17.0
**Acute Hepatitis B without Delta Agent**	B16.2, B16.9
**Acute Kidney Injury**	N17-, JVJF002, JVJF003, JVJF005, JVJF006, JVJF007, JVJF005, JVJB002
**Acute Respiratory Failure**	J96.0, J80-, J81-, GLLD002, GLLD003, GLLD017, GLLD019, GLLD004, GLLD005, GLLD006, GLLD007, GLLD008, GLLD009, GLLD012, GLLD013, GLLD015, DKMD001, GEPA004, GELD002, GELD004, ECCO2R, GLJF010
**AIDS**	B20-, B21-, B22-, B24-
**Alcohol Use Disorders**	E24.4, E51.1, F10.1, F10.2, F10.3, F10.4, F10.5, F10.6, F10.7, F10.8, F10.9, F10.2, F10.2, F10.2, F10.2, G31.2, G62.1, G72.1, I42.6, K29.2, K70-, K85.2, K86.0, O35.4, Z50.2, Z71.4, Z72.1
**Alcohol-Related Cirrhosis**	K70.3
**Alcohol-Related Hepatic Failure**	K70.4
**Alcohol-Related Hepatitis**	K70.1
**Alcohol-Related Liver Disease**	K70-
**Ascites**	R18-, HPHB003, HPJB001
**Aspergillosis**	B44-
**Autoimmune Hepatitis**	K75.4
**Bacteremia**	A49-
**Bronchopneumonia**	J18-
**Budd Chiari Syndrome**	I82.0
**Cerebral Infarction**	I63-
**Cerebrovascular Disease**	G45-, G46-, H34.0, I6
**Chemotherapy for Cancer**	Z51.1
**Cholangitis**	K83.0, K83.1, K83.5, K83.5, K87.0, R17-
**Chronic Hepatitis B**	B18.1
**Chronic Hepatitis C**	B18.2
**Chronic Hepatitis D**	B18.0
**Chronic Kidney Disease Advanced**	I12.0, I13.1, N00-, N01-, N03-, N05-, N18.3, N18.4, N18.5, N19-, N25.0, Z49.0, Z49.1, Z49.2, Z94.0, Z99.2, HGPC005, HPGA001, HPJP001, HPKA002, HPKB001, HPKC014, HPLA005, HPLB004, HPLC035, HPPA004, HPPP002, JVRP007, JVRP008, JAEA003,

	JVJB001, JVRP004, JVJF004, JVJF008, JVQF001, JVQF007, JVQP002, JVQP009, YYYY007
**Chronic Kidney Disease**	E10.2, E11.2, E12.2, E13.2, E14.2, I15.1, JAHB001, JAHH002, JAHC001, JAHA001, JAHJ006, JAHJ007, N02-, N04-, N06-, N07-, N08-, N18.1, N18.2, N19-, N25-, N08.3
**Chronic Obstructive Pulmonary Disease**	I27.8, I27.9, J40-, J41-, J42-, J43-, J44-, J45-, J46-, J47-, J60-, J61-, J62-, J63-, J64-, J65-, J66-, J67-, J68.4, J70.1, J70.3
**Cirrhosis**	I85.9, I86.4, I98.2, I98.2, K70.3, K71.7, K74.3, K74.4, K74.5, K74.6, K76.6
**Congestive Heart Failure**	I09.9, I11.0, I13.0, I13.2, I25.5, I42.0, I42.5, I42.6, I42.7, I42.8, I42.9, I43-, I50-, P29.0
**Connective Tissue Disorder**	M05-, M06-, M31.5, M32-, M33-, M34-, M35.1, M35.3, M36.0
**Cystic Fibrosis**	E84-
**Cystic Fibrosis with Pulmonary Manifestations**	E84.0
**Cystic Fibrosis with Intestinal Manifestations**	E84.1
**Cystic Fibrosis with Other Manifestations**	E84.8
**Cystic Fibrosis Unspecified**	E84.9
**Decompensated Cirrhosis**	R17-, K70.4, K71.1, K72-, K76.7, I28.0, R18-, J94.8, K65-, HPHB003, HPJB001, EHCA003, EHCA006, EHCA009, EHCA007, EHCA004, EHCA002, EHCA005, EHCA010, EHCA001, HEPA005, HEPA004, HEPA007
**Dementia**	F00-, F01-, F02-, F03-, F05.1, G30-, G31.1
**Diabetes Mellitus Complicated**	E10.2, E10.3, E10.4, E10.5, E10.7, E11.2, E11.3, E11.4, E11.5, E11.7, E12.2, E12.3, E12.4, E12.5, E12.7, E13.2, E13.3, E13.4, E13.5, E13.7, E14.2, E14.3, E14.4, E14.5, E14.7, H36.0, N08.3, H28.0, G63.2
**Diabetes Mellitus Uncomplicated**	E10.0, E10.1, E10.6, E10.8, E10.9, E11.0, E11.1, E11.6, E11.8, E11.9, E12.0, E12.1, E12.6, E12.8, E12.9, E13.0, E13.1, E13.6, E13.8, E13.9, E14.0, E14.1, E14.6, E14.8, E14.9
**Diabetes Mellitus**	E1
**Disseminated Intravascular Coagulation**	D65-, D68.4
**Gastro Esophageal Varices Bleeding**	I85.0, I98.3, I98.2, EHBD001, EHNE002, HESE001, HESE002
**Gastro Esophageal Varices not Bleeding**	I85.9, I86.4, I98.2, I98.2, EHNE001
**Hemiplegia**	G04.1, G11.4, G80.1, G80.2, G81-, G82-, G83.0, G83.1, G83.2, G83.3, G83.4, G83.9
**Hepatic Encephalopathy**	K72.0, G94.3, R40.2
**Hepatocellular Carcinoma Treatment**	YYYY170, EDLF017, EDLF016, HLNM001, EDQH006, EDLF014, YYYY210, HLHJ005, EDQH007, HLHJ006, EDLF015, EDQH008, HLNK001, EDSF006, EDLL001, ZZQA002, EHSF001, HLQX004, EDLL002, HLFA020

**Hepatocellular Carcinoma**	C22.0
**Hepatopulmonary Syndrome**	I28.0
**Hepatorenal Syndrome**	K76.7
**HIV Infection**	Z21-, B20-, B21-, B22-, B23-, B24-
**Hydrothorax**	J94.8
**Hypercholesterolemia Pure**	E87.0
**Hyperlipemia Mixed**	E78.2
**Hyperlipemia**	E78.0, E78.1, E78.2, E78.4, E78.5
**Hypertension**	I10-, I11-, I12-, I13-, R03.0
**Hypertensive Heart Failure with or without Renal Failure**	I10-, I13.0, I13.2
**Hypertriglyceridemia**	E87.1
**Intrahepatic Bile Duct Carcinoma**	C22.1
**Jaundice**	R17-
**Leukemia**	C91-, C92-, C93-, C94-, C95-, C96-
**Liver Biopsy**	HLHB001, HLHJ003, HLHJ006, HLHH006, HLHJ005, HLHH007, HLHH001, HLHH005
**Liver Disease Mild**	K71.3, K71.4, K71.5, K73-, K74.0, K74.1, K74.2, K74.3, K76.0, K76.4, K76.5
**Liver Disease Moderate to Severe**	K70.3, I85.0, I85.9, I86.4, I98.2, J94.8, K70.3, K70.4, K71.1, K71.7, K72.1, K72.9, K74.4, K74.5, K74.6, K65-, K66-, K76.7, R17-, R18-, EHBD001, EHNE002, HESE001, HESE002, HPHB003, HPJB001, EHCA003, EHCA006, EHCA009, EHCA007, EHCA004, EHCA002, EHCA005, EHCA010, EHCA001, HEPA005, HEPA004, HEPA007
**Liver Transplantation**	HLEA001, HGEA002, HLEA002, HGEA004
**Lung Transplantation**	GFEA004, GFEA001, GFEA006, GGBA001
**Lymphoma**	C81-, C82-, C83-, C84-, C85-, C86-, C87-, C89-, C90-
**Myocardial Infarction**	I21-, I22-, I25.2
**Obesity**	E66-
**Other Cause of Chronic Liver Disease**	E83.0, E83.1, FEJF003, I82.0, K83.0, K74.3, K75.4, Q44.2, Q44.3, Q44.6
**Other Primary Liver Cancer**	C22.2, C22.3, C22.4, C22.7, C22.9
**Palliative Care**	Z51.5
**Pancreatic Steatorrhoea**	K90-
**Pancreatitis Chronic Other**	K86.1
**Paraplegia Hemiplegia**	G04.1, G11.4, G80.1, G80.2, G81-, G82-, G83.0, G83.1, G83.2, G83.3, G83.4, G83.9
**Peptic Ulcer Disease**	K25-, K26-, K27-, K28-
**Peritonitis**	K65-
**Peripheral Vascular Disease**	I70-, I71-, I73.1, I73.8, I73.9, I77.1, I79.0, I79.2, K55.1, K55.8, K55.9, R02-, Z95.8, Z95.9
**Phlebitis and Thrombophlebitis**	I80-
**Pneumocystosis**	B59-

**Pneumonia Bacterial**	J13-, J14-, J15-, J16-, J17.0, J17.8, J18-, J85-
**Pneumonia Due to Streptococcus Pneumoniae**	J13-
**Pneumonia Due to Haemophilus Influenzae**	J14-
**Pneumonia Bacterial not Elsewhere Classified**	J15-
**Pneumonia Due to Other Infectious Organisms not Elsewhere Classified**	J16-
**Pneumonia in Diseases Classified Elsewhere**	J17.0, J17.8
**Portal Hypertension**	K76.6
**Portal Vein Thrombosis**	I81-
**Renal Transplantation**	T86.1, Z94.0, JAEA003
**Smoking**	F17-, Z71.6, Z72.0, T65.2
**Solid Tumor Localized without Liver**	C0, C1, C20-, C21-, C23-, C25-, C26-, C3, C40-, C41-, C43-, C45-, C46-, C47-, C48-, C49-, C5, C6, C70-, C71-, C72-, C73-, C74-, C75-, C76-
**Solid Tumor Localized**	C0, C1, C20-, C21-, C22-, C23-, C24-, C25-, C26-, C3, C40-, C41-, C43-, C45-, C46-, C47-, C48-, C49-, C5, C6, C70-, C71-, C72-, C73-, C74-, C75-, C76-
**Solid Tumor Metastatic**	C77-, C78-, C79-, C80-
**Splanchnic Thrombophlebitis**	I81-, I82-
**Surgical Shunt**	EHCA003, EHCA006, EHCA009, EHCA007, EHCA004, EHCA002, EHCA005, EHCA010, EHCA001, HEPA005, HEPA004, HEPA007
**Thrombophlebitis Migrans**	I82.1
**Transfusion**	FELF003, FELF004
**Transjugular Intrahepatic Posto Systemic Shunt**	EHCF002, EHAF004, EHPF001, EHNF001
**Transjugular Liver Biopsy**	HLHH001, HLHH005
**Transplant Recipient without Liver**	Z94.0, Z94.1, Z94.2, Z94.3, Z94.8, Z94.8, T86.1, T86.2, T86.3, T86.8, T86.8, T86.8, T86.8, Z94801, Z94802, Z94803, Z94804, Z94809, T86.0, JAEA003
**Type 1 Diabetes Mellitus**	E10-
**Type 2 Diabetes Mellitus**	E11-

**Table S2. Age at CFLD Progression and Competing Risks**				
**Event**	Exposure Era	Number of Events (%)	Median (IQR) Age at Event (Years)	*p* value
CFLD progression	**Pre-ETI**	**163 (6.6%)**	**26 (18 - 37)**	
	**Post-ETI**	**63 (0.8%)**	**28 (21 - 39.5)**	**0.177**
Gastroesophageal varices (non-bleeding)	**Pre-ETI**	**75 (3%)**	**20 (15 - 29)**	
	**Post-ETI**	**91 (1.2%)**	**19 (16 - 25.5)**	**0.599**
Gastroesophageal varices bleeding	**Pre-ETI**	**55 (2.2%)**	**25 (17 - 36.5)**	
	**Post-ETI**	**22 (0.3%)**	**24 (20 - 37)**	**0.604**
Decompensated cirrhosis	**Pre-ETI**	**126 (5.1%)**	**26 (19 - 37)**	
	**Post-ETI**	**46 (0.6%)**	**26.5 (20 - 36.8)**	**0.634**
Acute liver failure	**Pre-ETI**	**90 (3.6%)**	**29 (22 - 40)**	
	**Post-ETI**	**56 (0.7%)**	**27.5 (21.8 - 37)**	**0.537**
Hepatocellular carcinoma	**Pre-ETI**	**9 (0.4%)**	**56 (41 - 62)**	
	**Post-ETI**	**3 (0%)**	**58 (54 - 67)**	**0.46**
Liver transplantation	**Pre-ETI**	**26 (1.1%)**	**22.5 (18 - 29.5)**	
	**Post-ETI**	**2 (0%)**	**23 (22 - 24)**	**1**
Lung transplantation	**Pre-ETI**	**509 (20.6%)**	**28 (23 - 36)**	
	**Post-ETI**	**84 (1.1%)**	**30 (21 - 39)**	**0.856**
Death without transplantation	**Pre-ETI**	**242 (9.8%)**	**40 (27 - 65)**	
	**Post-ETI**	**185 (2.4%)**	**51 (36 - 73)**	**< 0.001**

This comparison captures temporal associations and does not reflect individual-level exposure to ETI. The ETI era commenced in France after December 2019. *p* values were computed using Wilcoxon Rank-Sum Test. Abbreviation: CFLD = cystic fibrosis liver disease; ETI = Elexacaftor, Tezacaftor, and Ivacaftor; IQR = interquartile range

**Table S3. Characteristics of pwCF, by CFLD Progression**				
Characteristic	Overall N = 3,814[1]	No CFLD Progression n = 3,697, 96.9%[1]	CFLD Progression n = 117, 3.07%[1]	*p* value[2]
**Censored during the ETI era**	1,907 (50.00%)	1,875 (50.72%)	32 (27.35%)	<0.001
**Age at censoring**	20 (18, 26)	20 (18, 25)	26 (19, 39)	<0.001
**Male sex**	2,021 (52.99%)	1,949 (52.72%)	72 (61.54%)	0.060
**Smoking habits**	190 (4.98%)	171 (4.63%)	19 (16.24%)	<0.001
**Alcohol use disorders**	89 (2.33%)	61 (1.65%)	28 (23.93%)	<0.001
**Liver risk factors**	52 (1.36%)	34 (0.92%)	18 (15.38%)	<0.001
**Type-2 diabetes mellitus**	799 (20.95%)	733 (19.83%)	66 (56.41%)	<0.001
**Obesity**	135 (3.54%)	123 (3.33%)	12 (10.26%)	<0.001
**HIV-infection**	7 (0.18%)	4 (0.11%)	3 (2.56%)	<0.001
**CCI ≥ 3**	415 (10.88%)	386 (10.44%)	29 (24.79%)	<0.001
**Deprivation quintiles 4 or 5**	1,485 (38.94%)	1,441 (38.98%)	44 (37.61%)	0.8
**Death without transplantation**	234 (6.14%)	207 (5.60%)	27 (23.08%)	<0.001
**Follow-up duration (months)**	34 (17, 54)	34 (17, 54)	36 (23, 54)	0.2

[1] n (%); Median (Q1, Q3)

[2] Pearson's Chi-squared test; Wilcoxon rank sum test; Fisher's exact test

CFLD progression was any of decompensated cirrhosis, portal hypertension bleeding, primary liver cancer, liver transplantation, or lung transplantation without prior CFLD progression. The Charlson Comorbidity Index was categorized into low (<3), intermediate (3–5), and high (>5) scores to reflect increasing levels of frailty. The French Deprivation Index was used to assess spatial socioeconomic and health inequalities, with scores divided into quintiles. The ETI era commenced in France after December 2019. Abbreviations: CCI = Charlson Comorbidity Index; CFLD = cystic fibrosis liver disease; ETI = Elexacaftor-Tezacaftor-Ivacaftor; FDEP = French Deprivation Index; pwCF = people with cystic fibrosis.

Fig. S1. Cumulative Incidence of CFLD Progression in pwCF Aged ≥12 Years by ETI era

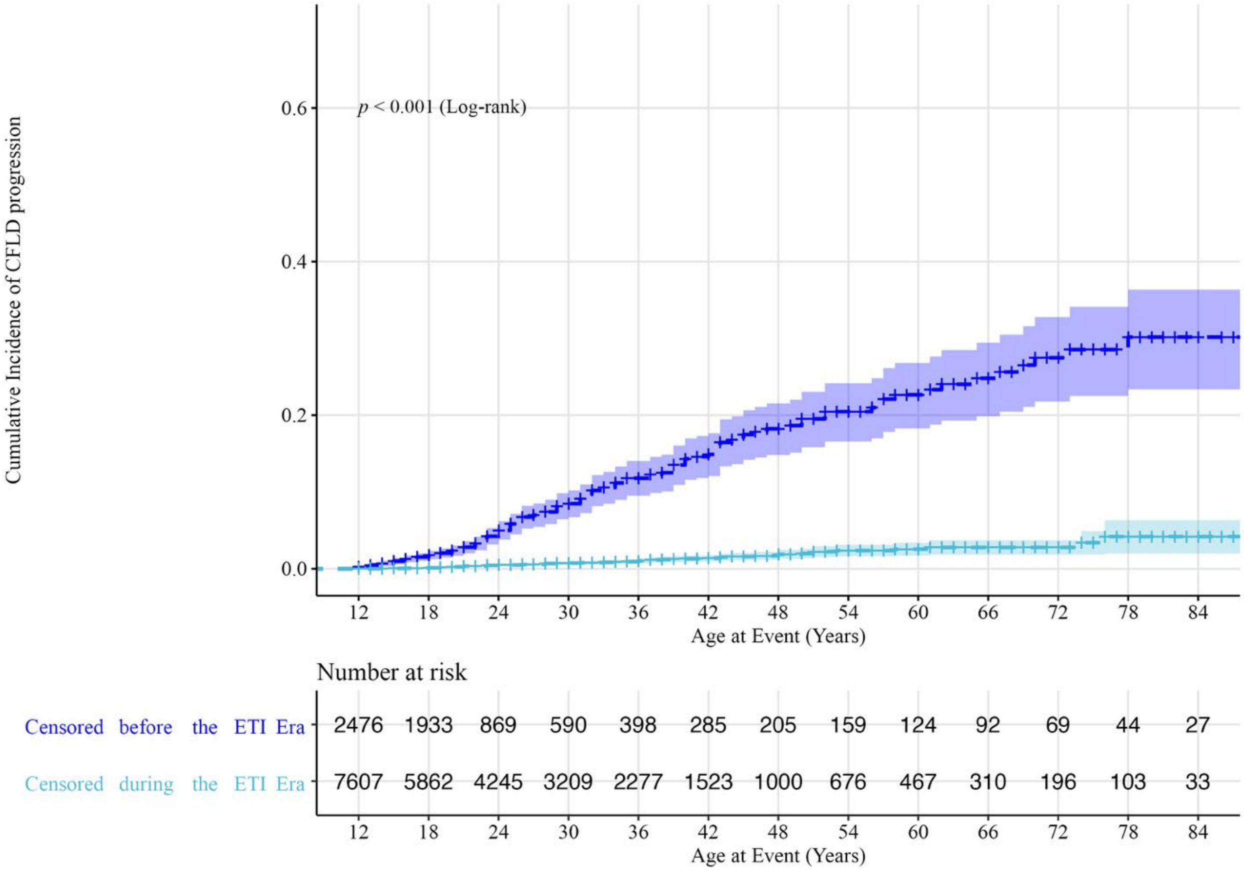

Kaplan–Meier estimates of CFLD progression probability in pwCF aged 12 years and older, stratified by whether they were censored before or after the introduction of ETI on December 15, 2019. This comparison reflects temporal trends but does not represent individual-level exposure to ETI. The total follow-up time was 75,276 person-months (6,273 person-years) in the pre-ETI era and 640,659 person-months in the post-ETI era (53,388 person-years). Abbreviations: CFLD = cystic fibrosis liver disease; ETI = Elexacaftor, Tezacaftor, and Ivacaftor, pwCF =people with cystic fibrosis.

Fig. S2. Covariate Balance Before and after Weighting in the Propensity Score–matched Sensitivity Analysis Cohort

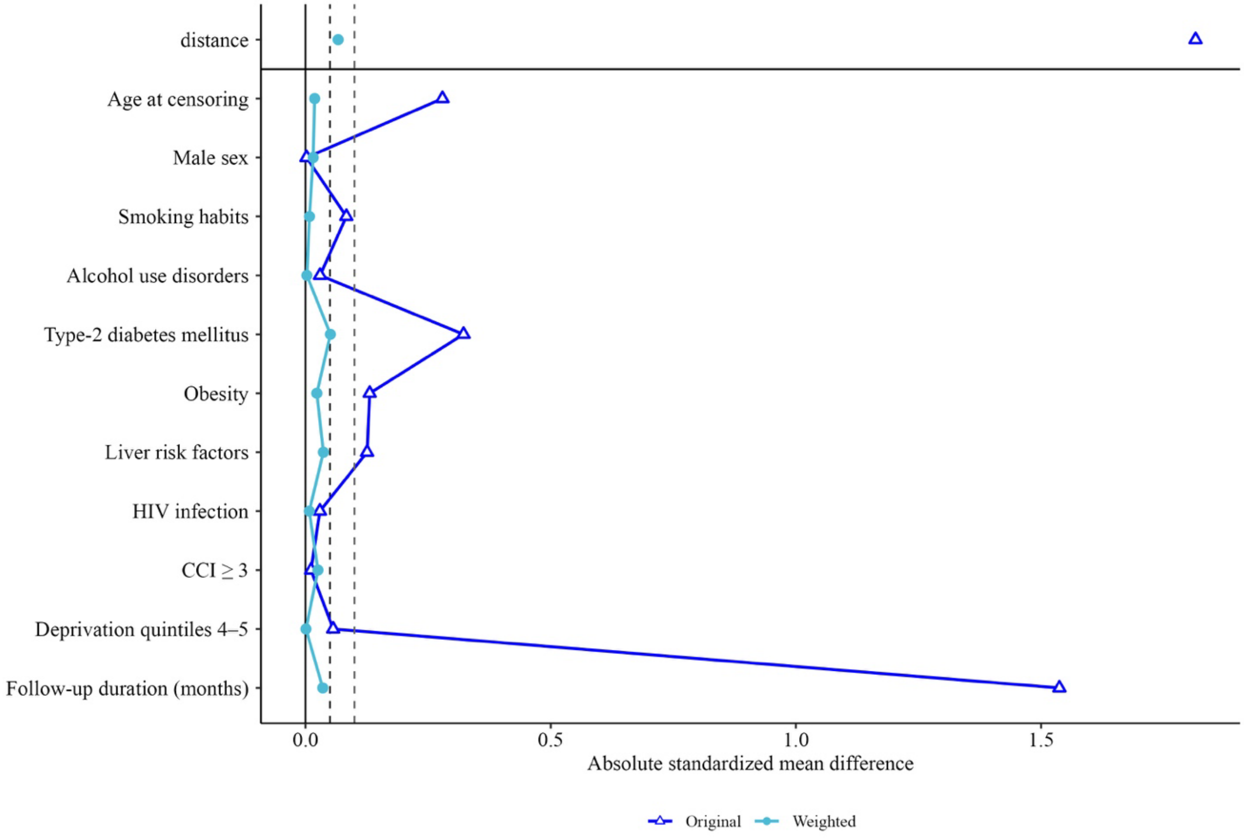

Propensity scores were estimated using logistic regression and matched by ETI period using a nearest-neighbor algorithm with a caliper of 0.1. Standardized mean differences for baseline characteristics before (unweighted) and after (weighted) propensity score matching among pwCF included in the analytic cohort. A threshold of 0.1 (dashed vertical line) denotes acceptable balance. Abbreviations: CCI = Charlson Comorbidity Index
