# Supplementary material for: Cystic fibrosis liver disease progression in the era of elexacaftor–tezacaftor–ivacaftor
Source: JHEP Rep. 2025 Jul 5;7(10):101512. doi: 10.1016/j.jhepr.2025.101512 (PMC12506492; doi:10.1016/j.jhepr.2025.101512)
Supplement: Multimedia component 4 [file mmc4.pdf]

JHEP|Reports

Cystic fibrosis liver disease progression in the era of elexacaftor–tezacaftor–ivacaftor

Authors

Charlotte Mouliade, Lucia Parlati, Stylianos Tzedakis, …, Philippe Sogni, Pierre-Régis Burgel, Vincent Mallet

Correspondence

vincent.mallet@aphp.fr (V. Mallet).

Graphical abstract

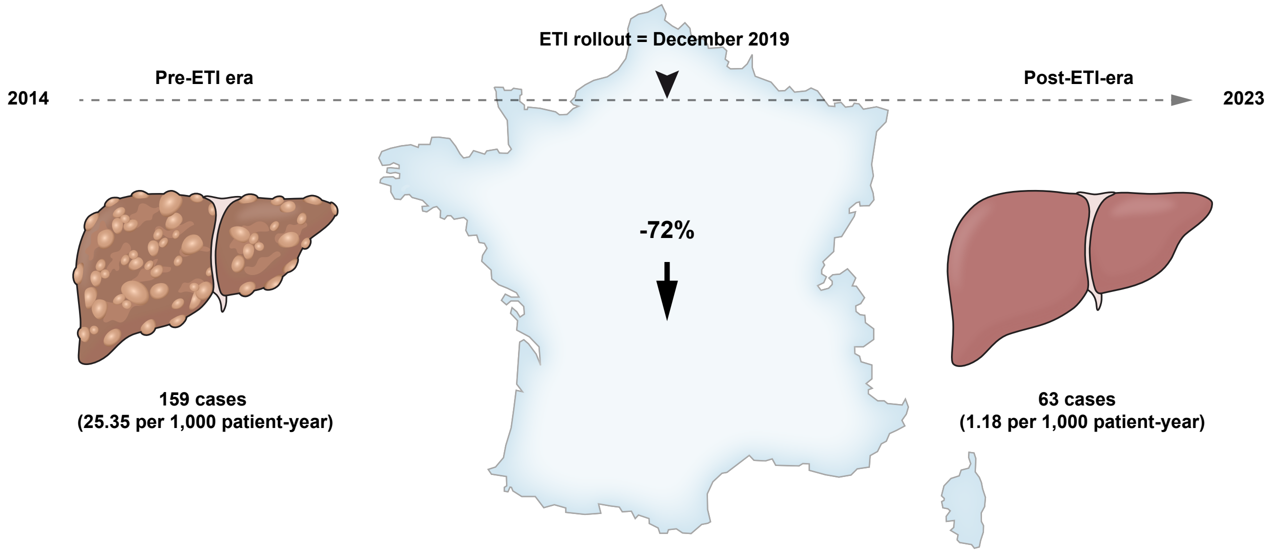

Highlights:

- Using nationwide French data, we evaluated liver disease progression among pwCF before and after ETI became available.
- Results showed 72% lower risk of severe liver complications after ETI introduction.
- Significant reductions in both overall mortality and the need for lung transplantation were observed following ETI rollout.
- ETI use was not associated with an increased risk of acute liver failure.
- These findings demonstrate a clear temporal association between ETI availability and improved liver outcomes in pwCF.

Impact and implications:

In this nationwide French cohort study of over 10,000 people with cystic fibrosis, we observed a significant decline in the incidence of cystic fibrosis liver disease outcomes following the rollout of elexacaftor–tezacaftor–ivacaftor (ETI) in December 2019. The rate dropped from 25.4 to 1.2 per 1,000 person-years post-ETI. Matched analyses confirmed a reduced risk of liver disease progression, with an adjusted incidence rate ratio of 0.28. Although the findings suggest ETI may improve liver outcomes in people with cystic fibrosis, potential confounding factors necessitate further research to establish causality and understand the underlying biological mechanisms.

https://doi.org/10.1016/j.jhepr.2025.101512
© 2025 The Author(s). Published by Elsevier B.V. on behalf of European Association for the Study of the Liver (EASL). This is an open access article under the CC BY license (http://creativecommons.org/licenses/by/4.0/).

Cystic fibrosis liver disease progression in the era of elexacaftor–tezacaftor–ivacaftor

Charlotte Mouliade[1,2], Lucia Parlati[1,2], Stylianos Tzedakis[2,3], Mathis Collier[4], Samir Bouam[5], Anais Vallet-Pichard[1], Valérie D'Halluin-Venier[1], Reem Kanaan[6], Stanislas Pol[1,2], Philippe Sogni[1,2,†], Pierre-Régis Burgel[2,6,†], Vincent Mallet[1,2,*], for the Demosthenes Research Group

JHEP Reports **2025**. vol. 7 | 1–7

Check for updates

Background & Aims: The effect of elexacaftor–tezacaftor–ivacaftor (ETI) on cystic fibrosis liver disease (CFLD) outcomes remains unknown. Thus, we investigated the association between the ETI rollout and trends in CFLD progression in a nationwide cohort.

Methods: Using the French National Hospital Discharge Database (2014–2023), we measured the incidence of CFLD progression (decompensated cirrhosis, gastroesophageal variceal bleeding, primary liver cancer, or liver transplantation) before and after ETI became available (December 2019) in patients with cystic fibrosis (pwCF) aged ≥12 years. Death without CFLD progression and lung transplantation were treated as competing events. Incidence rates were compared across ETI calendar eras in both the full cohort and a 1:1 propensity score-matched sample. Analyses used Kaplan–Meier curves, Fine–Gray competing risk models, and adjusted incidence rate ratios (aIRRs).

Results: The cohort included 10,083 pwCF (median age: 19 years [IQR 14–29]; 52.6% male), with 24.6% censored pre-ETI and 75.4% post-ETI. The overall incidence of CFLD progression was 3.7 per 1,000 person-years: 25.4 pre-ETI versus 1.2 post-ETI (p <0.001). The incidence of all CFLD outcomes, including non-bleeding varices, declined post-ETI (p <0.001). In matched analyses, pwCF censored during the ETI era had a lower incidence of CFLD progression (aIRR 0.28, 95% CI: 0.18–0.43; p <0.001). In addition, deaths in pwCF occurred at an older age during the ETI era.

Conclusions: The incidence of CFLD progression declined during the ETI rollout. While these findings suggest an association between ETI availability and improved liver outcomes, unmeasured confounders and concurrent changes in management might have also contributed. Thus, further studies are needed to confirm causality and understand underlying mechanisms.

© 2025 The Author(s). Published by Elsevier B.V. on behalf of European Association for the Study of the Liver (EASL). This is an open access article under the CC BY license (http://creativecommons.org/licenses/by/4.0/).

Introduction

Cystic fibrosis is an autosomal recessive disorder caused by variants in the gene encoding cystic fibrosis transmembrane conductance regulator (CFTR), leading to multisystem involvement, particularly in the lungs, gastrointestinal system, pancreas, and biliary tract.[1,2]

Cystic fibrosis liver disease (CFLD) is a complication of cystic fibrosis, affecting an estimated 20–40% of patients with cystic fibrosis (pwCF). Among these, 5–10% progress to severe liver disease, characterized by portal hypertension and/or decompensated cirrhosis.[3–6] Data from the Cystic Fibrosis Foundation Patient Registry (2003–2012) indicate 10-year cumulative rates of 6.6% for variceal bleeding, 9.9% for liver transplantation, and 6.9% for liver-related mortality,[7] noting that some pwCF undergo liver transplantation without CFLD progression to a liver-related complication.

The advent of CFTR modulators (Table 1), particularly the triple combination therapy elexacaftor–tezacaftor–ivacaftor (ETI), has revolutionized the management of cystic fibrosis.[2] ETI was initially developed in pwCF carrying at least one F508del variant, the most common CFTR variant worldwide, but it is also effective in pwCF carrying non-F508del variants, making this therapy suitable for up to 92% of pwCF.[8]

In France, ETI became available via an early-access program in December 2019. Over the subsequent 5 years, significant advances in managing pulmonary complications were documented.[2] However, the impact of ETI therapy on CFLD progression remains uncertain. Advanced liver disease was an exclusion criterion in all clinical trials evaluating CFTR modulators,[9–11] leaving a critical gap in understanding its effectiveness on CFLD outcomes. Consequently, current guidelines recommend neither for nor against CFTR modulator use in pwCF with CFLD.[12]

To address this evidence gap, we conducted a nationwide cohort study of pwCF hospitalized in France between 2014 and 2023. In the absence of individual-level data on ETI exposure, we adopted a calendar-based exposure definition, leveraging

* Corresponding author. Address: Assistance Publique-Hôpitaux de Paris, Hôpital Cochin, Service des Maladies du Foie, 27 rue du Faubourg Saint Jacques, 75014 Paris, France. Tel.: +33 1 58 41 30 01; fax: +33 1 58 41 30 15.
E-mail address: vincent.mallet@aphp.fr (V. Mallet).
† These authors contributed equally to this work.
https://doi.org/10.1016/j.jhepr.2025.101512

Table 1. Approved CFTR modulator treatments for pwCF.

Trade name	Active substance	ATC code	Filing date
Kalydeco	Ivacaftor	R07AX02	1 January 2012
Orkambi	Lumacaftor, ivacaftor	R07AX30	2 July 2015
Symkevi	Tezacaftor, ivacaftor	R07AX31	29 November 2018
Kaftrio*	ETI*	R07AX32*	7 August 2020

Source: European Medicines Agency.
CFTR, cystic fibrosis transmembrane conductance regulator; ETI, elexacaftor–tezacaftor–ivacaftor; pwCF, patients with cystic fibrosis.
*In France, ETI was made available via a nominative Temporary Use Authorization in December 2019.

the progressive rollout of ETI from December 2019 onward. Our objective was to compare the incidence of CFLD progression before and after the availability of ETI, and to generate population-level evidence for its potential hepatic benefit in pwCF.

Methods

Data source

Data were sourced from the French National Hospital Discharge Database (*Programme de Médicalisation des Systèmes d'Information; PMSI*). During the observational period, the entire French population (68.1 million in 2020) had equal access to universal, tax-financed healthcare services. The database contains anonymized, standardized discharge summaries that provide detailed patient demographics (age, sex, and postal code of residency), primary and associated discharge diagnoses coded using the WHO International Classification of Diseases, Tenth Revision (ICD-10), medical procedures, discharge dates, length of stay, and entry and discharge modes, including in-hospital mortality. Each patient's historical and underlying conditions were traced back to 2014 using unique anonymous identifiers.[13] The PMSI coding system has demonstrated 100% specificity for hard outcomes, including liver-related complications and in-hospital mortality. For sensitivity analyses and validation of our approach, see the study by Schwarzinger and colleagues.[14] Missingness was minimal (2.3% of deprivation score) for primary outcomes and key covariates. No imputation was performed; complete-case analyses were used. The study was approved by the *Commission Nationale de l'Informatique et des Libertés* under registration number DR-2017-404. We used deidentified data, and informed consent was not required.

Study population

We included all patients (ICD-10: E84%) aged 12 years or older with cystic fibrosis as a primary or associated discharge diagnosis between January 2014 and December 2023 (N = 12,625). To reduce the risk of false positivity, we retained pwCF with at least one primary discharge diagnosis of cystic fibrosis with pulmonary manifestations (E840, n = 9,833) or intestinal manifestations (E841, n = 1,165). pwCF who underwent liver transplantation without previous evidence of CFLD progression were excluded from the analysis.

Exposures

Individual-level data on ETI treatment initiation, genotype eligibility, and adherence are not available in the PMSI. Consequently, exposure was defined based on censoring during the ETI era, which began in France in December 2019 with the launch of the early-access program for pwCF aged ≥12 years. In the absence of individual treatment data, this calendar-based proxy was adopted to approximate ETI exposure. According to national registry data, ETI uptake increased progressively, with ~5.7% of eligible individuals treated in 2020 and 66.1% by 2023.[15] These estimates are based on the entire cystic fibrosis population and included ~900 lung-transplanted individuals and 400 children under the age of 2 years, who were not eligible for ETI. As a result, these uptake figures likely underestimate the true proportion of eligible patients who received ETI. Additional exposures included alcohol use disorders, smoking, chronic HBV or HCV, non-viral etiologies of chronic liver disease (*e.g.* congenital, autoimmune, or metabolic conditions), type 2 diabetes mellitus, obesity, major comorbidities, and socioeconomic deprivation. Definitions for alcohol use disorders, diabetes, and obesity are provided in the study by Mallet and colleagues.[16] Frailty was assessed using the Charlson Comorbidity Index (CCI), categorized as low (<3), intermediate (3–5), or high (>5). Socioeconomic status was evaluated using the French Deprivation Index, stratified into quintiles.[17,18] All covariates were coded as binary variables, except for age, which was modeled as a continuous variable. The complete codebook is provided in Table S1.

Outcome measure

The primary outcome was CFLD progression, defined as the first occurrence of decompensated cirrhosis (ascites, hepatic encephalopathy, or non-obstructive jaundice in patients with cirrhosis, excluding those with primary liver cancer), primary liver cancer (including hepatocellular carcinoma and intrahepatic cholangiocarcinoma), or gastroesophageal variceal bleeding. Gastroesophageal variceal bleeding without cirrhosis was observed in 31 cases (16.2%). Non-bleeding gastroesophageal varices were defined as the record of gastroesophageal varices or band ligation without a previous record of gastroesophageal variceal bleeding. We chose to censor pwCF at the time of lung transplantation, because this intervention can alter the natural course of CFLD. The secondary outcome was mortality.

Statistical analysis

Our objective was to compare the incidence rates and adjusted incidence rate ratios (aIRRs) of CFLD progression across the pre-ETI and ETI calendar periods. Given the gradual uptake of ETI and the absence of individual-level treatment exposure data, a causal inference framework was not

applicable. Therefore, we adopted a calendar-based exposure definition, anchored in the timeline of national ETI rollout. To mitigate confounding, we performed 1:1 propensity score matching (PSM) by ETI period (caliper 0.1), including follow-up duration as a covariate to adjust for potential lead-time bias, under the assumption that longer follow-up might reflect better underlying health. Covariate balance post matching is shown in Table S2. Incidence rates (per 1,000 person-years) were estimated and compared using Poisson regression with the logarithm of follow-up time as an offset, in both the unmatched and PSM samples. aIRRs were derived from multivariable Poisson models. All analyses were conducted using R (version 4.4.0, *Puppy Cup*; R Foundation for Statistical Computing, Vienna, Austria), with a two-sided significance threshold of p <0.05.

Results

pwCF cohort characteristics

The cohort included 10,083 pwCF aged ≥12 years, with a median age at inception of 19 years (IQR: 14–29) and a balanced sex distribution (52.6% male). Smoking and alcohol use disorders were recorded in 6.3% and 3.0% of patients, respectively. Liver-specific risk factors were present in 1.7% of the cohort. Diabetes mellitus or obesity affected 27.1% of patients. Overall, 24.6% were censored during the pre-ETI era and 75.4% during the ETI era. Patients censored during the ETI era were older at the time of censoring (p <0.001) and had longer follow-up durations (p <0.001). Smoking (p <0.001) and obesity (p <0.001) were more frequent in the ETI group, whereas liver risk factors (p <0.001), diabetes mellitus (p <0.001), and social deprivation (p = 0.016) were more prevalent in the pre-ETI group. Sex (p >0.9), comorbidity burden (p = 0.7), alcohol use disorders (p = 0.2), and HIV infection (p = 0.2) did not differ significantly between groups. Additional characteristics are detailed in Table 2.

Incidence of CFLD progression across ETI eras in the complete cohort

The cumulative incidence of CFLD progression (Fig. S1) was significantly lower among pwCF censored during the ETI era (p <0.001, Fine–Gray test). Overall, the incidence of CFLD progression was 3.7 per 1,000 person-years, decreasing from 25.4 during the pre-ETI era to 1.2 per 1,000 person-years during the post-ETI era (p <0.001). All liver-related events declined significantly during the ETI era (Table 3). The incidence of liver transplantation dropped from 4.7 to 0.04 per 1,000 person-years, whereas lung transplantation without previous CFLD progression decreased from 81.1 to 1.6 per 1,000 person-years (p <0.001 for both). Records of non-bleeding gastroesophageal varices and/or band ligations were also significantly less common during the post-ETI era (p <0.001). Acute liver failure did not increase during the post-ETI era (p <0.001). Overall mortality declined more than 15-fold (p <0.001). Despite the marked reduction in liver-related outcomes, the median age at CFLD progression did not differ significantly between eras, whereas the age at death was ∼10 years higher during the post-ETI era (p <0.001; Table S2).

Incidence of CFLD progression across ETI eras in matched patients

The cumulative incidence of CFLD progression in the PSM cohort by ETI period (n = 3,814; see Fig. S2 for covariate balance) was significantly lower among pwCF censored during the ETI era (p <0.001, Fine–Gray test; Fig. 1). Follow-up duration was not associated with the risk of CFLD progression in the matched cohort (p = 0.2). Other risks for CFLD progression are detailed in Table S3. In the corresponding multivariable analysis, censoring during the ETI era remained independently associated with a significantly lower incidence of CFLD progression, with an aIRR of 0.28 (95% CI: 0.18–0.43; p <0.001). Other independent risk factors included alcohol use disorders

Table 2. Characteristics of pwCF aged 12 years and older across ETI eras.

Characteristic	Overall N = 10,083*	Censored during pre-ETI era (n = 2,476, 24.6%)*	Censored during ETI era (n = 7,607, 75.4%)*	p value†
Age at inception (years)	19 (14, 29)	18 (14, 26)	19 (12, 30)	0.083
Age at censoring (years)	24 (18, 36)	20 (18, 29)	26 (18, 38)	<0.001
Male sex	5,301 (52.57)	1,302 (52.58)	3,999 (52.57)	>0.9
Smoking habits	634 (6.29)	117 (4.73)	517 (6.80)	<0.001
Alcohol use disorders	308 (3.05)	65 (2.63)	243 (3.19)	0.2
Liver risk factors	167 (1.66)	67 (2.71)	100 (1.31)	<0.001
Chronic HBV	37 (0.37)	12 (0.48)	25 (0.33)	0.3
Chronic HCV	37 (0.37)	18 (0.73)	19 (0.25)	<0.001
Chronic HDV	4 (0.04)	2 (0.08)	2 (0.03)	0.3
Non-viral causes of chronic liver disease	106 (1.05)	46 (1.86)	60 (0.79)	<0.001
Diabetes mellitus	2,341 (23.22)	808 (32.63)	1,533 (20.15)	<0.001
Obesity	582 (5.77)	81 (3.27)	501 (6.59)	<0.001
HIV infection	39 (0.39)	6 (0.24)	33 (0.43)	0.2
CCI ≥3	1,416 (14.04)	341 (13.77)	1,075 (14.13)	0.7
Deprivation quintile 4 or 5	3,710 (37.69)	960 (39.75)	2,750 (37.02)	0.016
Follow-up duration (months)	78 (35, 110)	29 (12, 47)	97 (63, 114)	<0.001

This comparison captures temporal associations and does not reflect individual-level exposure to ETI. The ETI era started in France after December 2019. Patients were censored at the time of CFLD progression or upon undergoing lung transplantation without previous CFLD progression. The CCI was categorized into low (<3), intermediate (3–5), and high (>5) scores to reflect increasing levels of frailty. The French Deprivation Index was used to assess spatial socioeconomic and health inequalities, with scores divided into quintiles. CCI, Charlson Comorbidity Index; ETI, elexacaftor–tezacaftor–ivacaftor; FDEP, French Deprivation Index.

*Data are presented as median (Q1, Q3) or n (%).

†Wilcoxon rank sum test; Pearson's Chi-square test; Fisher's exact test.

Table 3. Incidence of CFLD progression in pwCF aged 12 years and older across ETI eras.

Event	Overall*	Pre-ETI incidence*	Post-ETI incidence*	Adjusted IRR (95% CI)	*p* value
CFLD progression	222 (3.72)	159 (25.35)	63 (1.18)	0.06 (0.05–0.08)	<0.001
Gastroesophageal varices (non-bleeding)	166 (2.78)	75 (11.96)	91 (1.7)	0.22 (0.15–0.3)	<0.001
Gastroesophageal varices (bleeding)	77 (1.29)	55 (8.77)	22 (0.41)	0.06 (0.04–0.11)	<0.001
Decompensated cirrhosis	172 (2.88)	126 (20.09)	46 (0.86)	0.06 (0.04–0.09)	<0.001
Hepatocellular carcinoma	12 (0.2)	9 (1.43)	3 (0.06)	0.03 (0.01–0.13)	<0.001
Acute liver failure	146 (2.45)	90 (14.35)	56 (1.05)	0.08 (0.06–0.12)	<0.001
Liver transplantation	28 (0.47)	26 (4.14)	2 (0.04)	0.02 (0–0.08)	<0.001
Lung transplantation	593 (9.94)	509 (81.14)	84 (1.57)	0.02 (0.02–0.03)	<0.001
Overall mortality	565 (9.47)	370 (58.98)	195 (3.65)	0.06 (0.05–0.07)	<0.001

The total follow-up time was 75,276 person-months (6,273 person-years) during the pre-ETI era and 640,659 person-months during the post-ETI era (i.e. 53,388 person-years). aIRRs and *p* values were estimated from Poisson regression models adjusted for age, sex, smoking, alcohol use disorders, liver and metabolic risk factors, major comorbidities, and social deprivation with an offset for log(person-time). This comparison captures temporal associations and does not reflect individual-level exposure to ETI.
aIRRs, adjusted incidence rate ratios; CFLD, cystic fibrosis liver disease; ETI, elexacaftor–tezacaftor–ivacaftor.
*Data are presented as *n* (%).

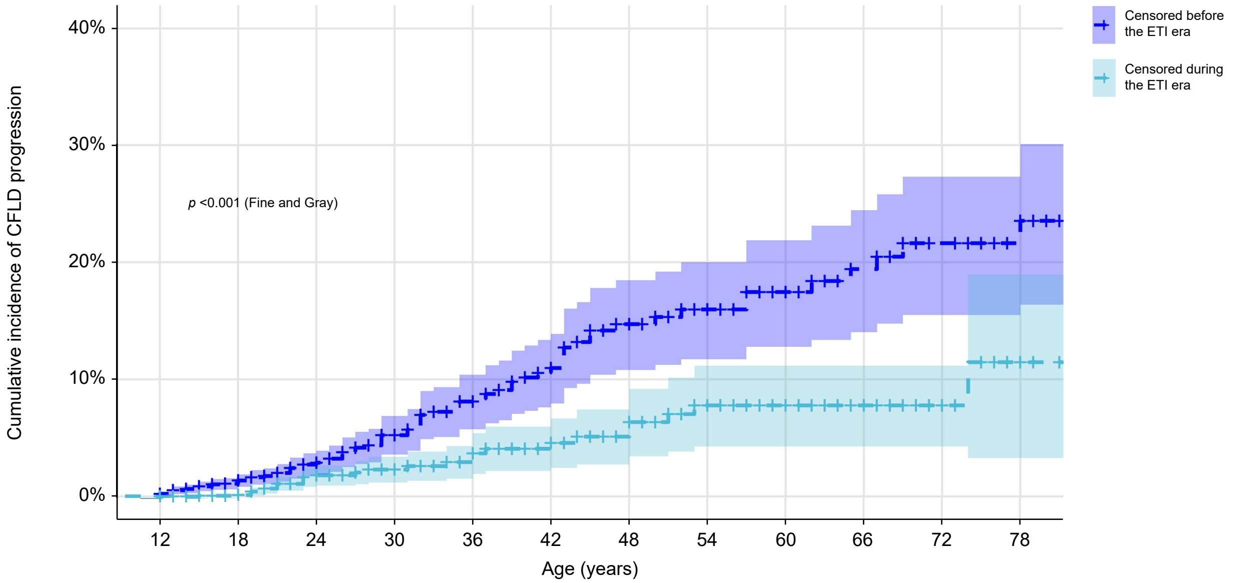

No. at risk												
Censored before the ETI Era	1,907	1,464	616	420	295	213	153	124	95	76	60	41
Censored during the ETI Era	1,907	1,600	588	345	261	193	153	116	93	55	34	16

Fig. 1. Cumulative incidence of CFLD progression in matched pwCF aged ≥12 years across ETI eras. Kaplan–Meier estimates of CFLD progression probability in pwCF aged 12 years and older, stratified by whether they were censored before or after the introduction of ETI on December 15, 2019. This comparison reflects temporal trends, but does not represent individual-level exposure to ETI. CFLD progression was any of decompensated cirrhosis, portal hypertension bleeding, primary liver cancer, liver transplantation, or lung transplantation without previous CFLD progression. CFLD, cystic fibrosis liver disease; ETI, elexacaftor–tezacaftor–ivacaftor; pwCF, patients with cystic fibrosis.

(*p* <0.001), diabetes mellitus (*p* <0.001), and liver-related comorbidities (*p* = 0.001) (Fig. 2).

Discussion

In this nationwide analysis, we observed a marked reduction in the incidence of CFLD progression and all-cause in-hospital mortality among French pwCF aged ≥12 years during the period following the introduction of ETI. In a PSM cohort designed to reduce measured confounding, censoring during the ETI era was associated with a >70% lower incidence rate of CFLD progression (aIRR 0.28, 95% CI: 0.18–0.43; *p* <0.001). Notably, the incidence of acute liver failure did not increase during the post-ETI period.

This study addresses a key knowledge gap identified in recent guidelines for the management of hepatobiliary complications in pwCF.[12] To our knowledge, it provides the first nationwide population-based analysis of CFLD outcomes across the ETI rollout period. The observed reduction in severe liver-related events during this timeframe suggests that ETI has a role in modifying the hepatic trajectory of pwCF. However, in the absence of individual treatment data, this association remains hypothesis generating.

Our findings align with small observational studies reporting improved liver parameters under CFTR modulator therapy.[19–24] Although reports on liver stiffness changes remain inconsistent,[19,25] reductions in liver transplantation rates in our study mirror trends reported in UNOS registry data,[26] and parallel the decline in lung transplantation rates observed in the USA post-ETI rollout.[27] Importantly, we found no evidence of increased risk of acute liver failure during the ETI era, consistent with pediatric safety reports.[28]

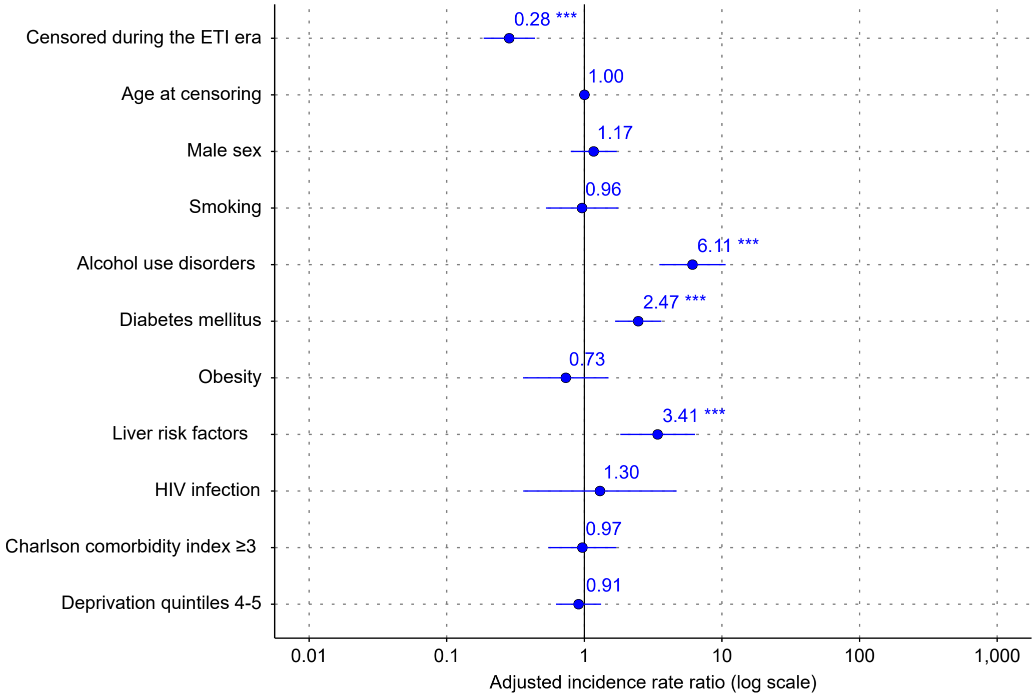

Fig. 2. Adjusted incidence rate ratios for CFLD progression in matched pwCF aged ≥12 years across ETI eras. Adjusted incidence rate ratios were estimated from a weighted multivariable Poisson regression model assessing the association between censoring before or after the introduction of ETI (15 December 2019) and CFLD progression. Time at risk was included as a log-transformed offset term. Results are based on the propensity score-matched sample by ETI period (see Fig. S2 for covariate balance). CFLD, cystic fibrosis liver disease; ETI, elexacaftor–tezacaftor–ivacaftor; pwCF, patients with cystic fibrosis.

CFTR is expressed in the biliary epithelium, where dysfunction alters bile secretion and flow, contributing to cholangiopathy and eventually portal hypertension.[29,30] ETI improves CFTR function and might thereby enhance bile fluidity and flow, potentially reducing biliary obstruction, inflammation, and fibrosis.[31] Although the biliary phenotype of CFLD could plausibly respond to CFTR modulation, the vascular subtype, characterized by non-cirrhotic portal hypertension, has an uncertain pathogenesis and its response to ETI is unknown.

Although the observed decrease in portal hypertension-related complications during the ETI era is consistent with a plausible biological mechanism, this interpretation should be viewed with caution. Changes in clinical practice, supportive care, nutritional status, or hospitalization thresholds over time could also account for some of the observed trend. Furthermore, our reliance on calendar time as a proxy for ETI exposure limits any causal interpretation. The reduction in non-liver-related outcomes (*e.g.* lung transplantation and mortality) might reflect overall improvements in care, beyond the specific hepatic effects of ETI.

Our study has limitations. First, individual-level data on ETI initiation, duration, adherence, and genotype eligibility were not available, precluding any direct inference about drug efficacy. We also had no information on previous CFTR modulator therapies. Second, we relied on discharge diagnoses to identify outcomes, which can be subject to under- or over-coding. However, the specificity of PMSI coding for severe liver-related complications is high, and our outcome definitions were conservative. Third, we were unable to capture subclinical or early-stage liver disease, which might precede overt CFLD events. Fourth, although PSM was used to balance observed covariates, unmeasured confounding, such as nutritional status, adherence to supportive treatments, or evolving care standards, might have contributed to the findings. The gradual ETI uptake,[15] together with differential survival, introduces the risk of immortal time bias, although we accounted for follow-up duration in all models. Finally, the use of a calendar-based exposure design, although necessitated by data limitations, restricted our ability to emulate a target trial and prevented the use of causal language.

In summary, we report a temporal association between the ETI era and a lower burden of CFLD progression and mortality in pwCF at the national level. Although these findings might reflect a hepatic benefit of ETI, they should be interpreted as preliminary and exploratory. The results do not establish causality, but do provide a strong rationale for further research. Future prospective studies should aim to combine clinical, biological, and imaging data to better characterize early hepatic changes under ETI and distinguish between subtypes of CFLD. Individual-level data on ETI exposure will be crucial to clarify treatment effects and inform personalized care. Until such data are available, our study offers important population-level insights that could inform clinical vigilance, guideline development, and research priorities in the management of CFLD during the ETI era.

Affiliations

[1]AP-HP Centre Université Paris Centre, Groupe Hospitalier Cochin Port Royal, DMU Cancérologie et Spécialités Médico-Chirurgicales, Service des Maladies du foie, Paris, France; [2]Université Paris Cité, F-75006, Paris, France; [3]AP-HP Centre, Groupe Hospitalier Cochin Port Royal, DMU Cancérologie et Spécialités Médico-Chirurgicales, Service de Chirurgie Digestive Hépatobiliaire et Endocrinienne, Paris, France; [4]AP-HP Centre, Groupe Hospitalier Cochin Port Royal, DMU Prime, Unité de Recherche Clinique, Paris, France; [5]AP-HP Centre Université de Paris, Groupe Hospitalier Cochin Port Royal, DMU PRIM, Service d'Information Médicale, Paris, France; [6]AP-HP Centre, Groupe Hospitalier Cochin Port Royal, DMU Thoros, Service de Pneumologie et Centre National de Référence de la Mucoviscidose, Paris, France

Abbreviations

aIRRs, adjusted incidence rate ratios; CCI, Charlson Comorbidity Index; CFLD cystic fibrosis liver disease; CFTR, cystic fibrosis transmembrane conductance regulator; ETI, ,elexacaftor–tezacaftor–ivacaftor; FDEP, French Deprivation Index; ICD-10, International Classification of Diseases, Tenth Revision; PMSI, Programme de Médicalisation des Systèmes d'Information; PSM, propensity score matching; pwCF, patients with cystic fibrosis.

Financial support

The study did not receive any external funding.

Conflicts of interest

VM reports a role as a coinvestigator for Genfit, Intercept, Janssen, Novo-Nordisk, and Galmed; and travel fees from AbbVie. SP reports consulting and lecturing fees from Gilead, Abbvie, NovoNordisk, Pfizer, Viiv, and LFB, and grants from Gilead and Abbvie. PRB reports consulting fees from Astra Zeneca, Chiesi, Insmed, Pfizer, Sanofi, MSD, Viatris, and Vertex; and travel fees Astra Zeneca, Chiesi. The remaining authors have no conflicts of interest to report.

Please refer to the accompanying ICMJE disclosure forms for further details.

Authors' contributions

Conception of the study, analysis and interpretation of the data, drafting of manuscript: VM. Analysis and interpretation of the data, editing of manuscript: CM, LP, ST, MC, PS, SB, RK, PRB. Acquisition of data, editing of manuscript: SB. Statistical analysis supervision: MC. Responsible for the content of the report: VM. All authors have seen and approved the final version of the manuscript.

Data availability

None.

Supplementary data

Supplementary data to this article can be found online at https://doi.org/10.1016/j.jhepr.2025.101512.

References

Author names in bold designate shared co-first authorship

[1] Grasemann H, Ratjen F. Cystic fibrosis. N Engl J Med 2023;389:1693–1707.
[2] Mall MA, Burgel PR, Castellani C, et al. Nat Rev Dis Primers 2024;10:53.
[3] Colombo C, Battezzati PM, Crosignani A, et al. Liver disease in cystic fibrosis: a prospective study on incidence, risk factors, and outcome. Hepatology 2002;36:1374–1382.
[4] Lamireau T, Monnereau S, Martin S, et al. Epidemiology of liver disease in cystic fibrosis: a longitudinal study. J Hepatol 2004;41:920–925.
[5] Scott-Jupp R, Lama M, Tanner MS. Prevalence of liver disease in cystic fibrosis. Arch Dis Child 1991;66:698–701.
[6] Chryssostalis A, Hubert D, Coste J, et al. Liver disease in adult patients with cystic fibrosis: a frequent and independent prognostic factor associated with death or lung transplantation. J Hepatol 2011;55:1377–1382.
[7] Ye W, Narkewicz MR, Leung DH, et al. Variceal hemorrhage and adverse liver outcomes in patients with cystic fibrosis cirrhosis. J Pediatr Gastroenterol Nutr 2018;66:122–127.
[8] Burgel PR, Sermet-Gaudelus I, Girodon E, et al. The expanded French compassionate programme for elexacaftor-tezacaftor-ivacaftor use in people with cystic fibrosis without a F508del CFTR variant: a real-world study. Lancet Respir Med 2024;12:888–900.
[9] Wainwright CE, Elborn JS, Ramsey BW. Lumacaftor-ivacaftor in patients with cystic fibrosis homozygous for Phe508del CFTR. N Engl J Med 2015;373:1783–1784.
[10] Ratjen F, Hug C, Marigowda G, et al. Efficacy and safety of lumacaftor and ivacaftor in patients aged 6-11 years with cystic fibrosis homozygous for F508del-CFTR: a randomised, placebo-controlled phase 3 trial. Lancet Respir Med 2017;5:557–567.
[11] **Middleton PG**, **Mall MA**, Drevinek P, et al. Elexacaftor-tezacaftor-ivacaftor for cystic fibrosis with a single Phe508del allele. N Engl J Med 2019;381:1809–1819.
[12] Sellers ZM, Assis DN, Paranjape SM, et al. Cystic fibrosis screening, evaluation, and management of hepatobiliary disease consensus recommendations. Hepatology 2024;79:1220–1238.
[13] Agence Technique de l'Information sur l'Hospitalisation. Aide à l'utilisation des informations de chainage. www.atih.sante.fr/aide-lutilisation-des-informations-de-chainage Accessed 10 July 2025.
[14] Schwarzinger M, Baillot S, Yazdanpanah Y, et al. Contribution of alcohol use disorders on the burden of chronic hepatitis C in France, 2008-2013: a nationwide retrospective cohort study. J Hepatol 2017;67:454–461.
[15] Vaincre la Mucoviscidose & Ined. Registre Français de la Mucoviscidose – Bilan des données 2023. www.vaincrelamuco.org/face-la-mucoviscidose/registre-et-muco-en-chiffres/valorisation-des-donnees Accessed 10 July 2025.
[16] Mallet V, Parlati L, Martinino A, et al. Burden of liver disease progression in hospitalized patients with type 2 diabetes mellitus. J Hepatol 2022;76:265–274.
[17] Quan H, Li B, Couris CM, et al. Updating and validating the Charlson comorbidity index and score for risk adjustment in hospital discharge abstracts using data from 6 countries. Am J Epidemiol 2011;173:676–682.
[18] Rey G, Jougla E, Fouillet A, et al. Ecological association between a deprivation index and mortality in France over the period 1997 - 2001: variations with spatial scale, degree of urbanicity, age, gender and cause of death. BMC Public Health 2009;9:33.
[19] Calvo PL, Giugliano L, Evangelista A, et al. Effects of CFTR modulator therapies on liver stiffness and bile flow: a single-centre experience. J Hepatol 2023;79:e76–e78.
[20] Drummond D, Dana J, Berteloot L, et al. Lumacaftor-ivacaftor effects on cystic fibrosis-related liver involvement in adolescents with homozygous F508 del-CFTR. J Cyst Fibros 2022;21:212–219.
[21] Gelzo M, Iacotucci P, Caputo M, et al. Lumacaftor/ivacaftor improves liver cholesterol metabolism but does not influence hypocholesterolemia in patients with cystic fibrosis. J Cyst Fibros 2021;20:e1–e6.
[22] Kutney K, Donnola SB, Flask CA, et al. Lumacaftor/ivacaftor therapy is associated with reduced hepatic steatosis in cystic fibrosis patients. World J Hepatol 2019;11:761–772.
[23] Levitte S, Fuchs Y, Wise R, et al. Effects of CFTR modulators on serum biomarkers of liver fibrosis in children with cystic fibrosis. Hepatol Commun 2023;7:e0010.
[24] Ramsey ML, Wellner MR, Porter K, et al. Cystic fibrosis patients on cystic fibrosis transmembrane conductance regulator modulators have a reduced incidence of cirrhosis. World J Hepatol 2022;14:411–419.
[25] Schnell A, Jungert J, Klett D, et al. Increase of liver stiffness and altered bile acid metabolism after triple CFTR modulator initiation in children and young adults with cystic fibrosis. Liver Int 2023;43:878–887.
[26] **Mendizabal M**, **Cywes C**, **Munoz N**, et al. Have CFTR modulators changed the need for liver and lung transplantation among patients with cystic fibrosis? An analysis of the UNOS database. Liver Transpl 2025;31:412–416.
[27] Pena TA, Wright B, Parekh KR, et al. Impact of elexacaftor-tezacaftor-ivacaftor in lung transplantation for cystic fibrosis in the United States. JHLT Open 2025;7:100171.

[28] Protich HE, Molleston JP, Bozic M, et al. Elexacaftor/tezacaftor/ivacaftor use in pediatric cystic fibrosis patients with advanced liver disease. J Cyst Fibros 2024;23:1122–1128.
[29] Fiorotto R, Strazzabosco M. Pathophysiology of cystic fibrosis liver disease: a channelopathy leading to alterations in innate immunity and in microbiota. Cell Mol Gastroenterol Hepatol 2019;8:197–207.
[30] Wilschanski M, Durie PR. Patterns of GI disease in adulthood associated with mutations in the CFTR gene. Gut 2007;56:1153–1163.
[31] **van de Peppel IP**, **Doktorova M**, Berkers G, et al. IVACAFTOR restores FGF19 regulated bile acid homeostasis in cystic fibrosis patients with an S1251N or a G551D gating mutation. J Cyst Fibros 2019;18:286–293.

Keywords: Cystic fibrosis; Drug therapy; Chloride channel agonists; Therapeutic use; Liver diseases; Genetics; Mortality; Longitudinal study; Cohort study.
Received 27 December 2024; received in revised form 10 June 2025; accepted 30 June 2025; Available online 5 July 2025

Journal of Hepatology, Volume *7*

Supplemental information

Cystic fibrosis liver disease progression in the era of elexacaftor–teza-caftor–ivacaftor

Charlotte Mouliade, Lucia Parlati, Stylianos Tzedakis, Mathis Collier, Samir Bouam, Anais Vallet-Pichard, Valérie D'Halluin-Venier, Reem Kanaan, Stanislas Pol, Philippe Sogni, Pierre-Régis Burgel, Vincent Mallet, and for the Demosthenes Research Group

Cystic fibrosis liver disease progression in the era of elexacaftor–tezacaftor–ivacaftor

Charlotte Mouliade, Lucia Parlati, Stylianos Tzedakis, Mathis Collier, Samir Bouam, Anais Vallet-Pichard, Valérie D'Halluin-Venier, Reem Kanaan, Stanislas Pol, Philippe Sogni, Pierre-Régis Burgel, Vincent Mallet, for the Demosthenes research group

Table of contents

Other members of the Demosthenes Group 2

Table S1 3

Table S2 7

Table S3 8

Fig. S1 9

Fig. S2 10

Other members of the Demosthenes Group

Marion Corouge,1 Daniel Karinthi, 1 for the Demosthenes research group

1. AP-HP.Centre Université Paris Centre, Groupe Hospitalier Cochin Port Royal, DMU Cancérologie et spécialités médico-chirurgicales, Service des Maladies du Foie, Paris, France

**Table S1: Code Dictionary**	
**Diagnosis**	ICD-10/medical procedure codes
**Acute and Subacute Hepatic Failure**	K72.0, K71.2
**Acute Hepatitis a with Hepatic Coma**	B15.0
**Acute Hepatitis a without Hepatic Coma**	B15.9
**Acute Hepatitis B with Delta Agent**	B16.0, B16.1, B17.0
**Acute Hepatitis B without Delta Agent**	B16.2, B16.9
**Acute Kidney Injury**	N17-, JVJF002, JVJF003, JVJF005, JVJF006, JVJF007, JVJF005, JVJB002
**Acute Respiratory Failure**	J96.0, J80-, J81-, GLLD002, GLLD003, GLLD017, GLLD019, GLLD004, GLLD005, GLLD006, GLLD007, GLLD008, GLLD009, GLLD012, GLLD013, GLLD015, DKMD001, GEPA004, GELD002, GELD004, ECCO2R, GLJF010
**AIDS**	B20-, B21-, B22-, B24-
**Alcohol Use Disorders**	E24.4, E51.1, F10.1, F10.2, F10.3, F10.4, F10.5, F10.6, F10.7, F10.8, F10.9, F10.2, F10.2, F10.2, F10.2, G31.2, G62.1, G72.1, I42.6, K29.2, K70-, K85.2, K86.0, O35.4, Z50.2, Z71.4, Z72.1
**Alcohol-Related Cirrhosis**	K70.3
**Alcohol-Related Hepatic Failure**	K70.4
**Alcohol-Related Hepatitis**	K70.1
**Alcohol-Related Liver Disease**	K70-
**Ascites**	R18-, HPHB003, HPJB001
**Aspergillosis**	B44-
**Autoimmune Hepatitis**	K75.4
**Bacteremia**	A49-
**Bronchopneumonia**	J18-
**Budd Chiari Syndrome**	I82.0
**Cerebral Infarction**	I63-
**Cerebrovascular Disease**	G45-, G46-, H34.0, I6
**Chemotherapy for Cancer**	Z51.1
**Cholangitis**	K83.0, K83.1, K83.5, K83.5, K87.0, R17-
**Chronic Hepatitis B**	B18.1
**Chronic Hepatitis C**	B18.2
**Chronic Hepatitis D**	B18.0
**Chronic Kidney Disease Advanced**	I12.0, I13.1, N00-, N01-, N03-, N05-, N18.3, N18.4, N18.5, N19-, N25.0, Z49.0, Z49.1, Z49.2, Z94.0, Z99.2, HGPC005, HPGA001, HPJP001, HPKA002, HPKB001, HPKC014, HPLA005, HPLB004, HPLC035, HPPA004, HPPP002, JVRP007, JVRP008, JAEA003,

	JVJB001, JVRP004, JVJF004, JVJF008, JVQF001, JVQF007, JVQP002, JVQP009, YYYY007
**Chronic Kidney Disease**	E10.2, E11.2, E12.2, E13.2, E14.2, I15.1, JAHB001, JAHH002, JAHC001, JAHA001, JAHJ006, JAHJ007, N02-, N04-, N06-, N07-, N08-, N18.1, N18.2, N19-, N25-, N08.3
**Chronic Obstructive Pulmonary Disease**	I27.8, I27.9, J40-, J41-, J42-, J43-, J44-, J45-, J46-, J47-, J60-, J61-, J62-, J63-, J64-, J65-, J66-, J67-, J68.4, J70.1, J70.3
**Cirrhosis**	I85.9, I86.4, I98.2, I98.2, K70.3, K71.7, K74.3, K74.4, K74.5, K74.6, K76.6
**Congestive Heart Failure**	I09.9, I11.0, I13.0, I13.2, I25.5, I42.0, I42.5, I42.6, I42.7, I42.8, I42.9, I43-, I50-, P29.0
**Connective Tissue Disorder**	M05-, M06-, M31.5, M32-, M33-, M34-, M35.1, M35.3, M36.0
**Cystic Fibrosis**	E84-
**Cystic Fibrosis with Pulmonary Manifestations**	E84.0
**Cystic Fibrosis with Intestinal Manifestations**	E84.1
**Cystic Fibrosis with Other Manifestations**	E84.8
**Cystic Fibrosis Unspecified**	E84.9
**Decompensated Cirrhosis**	R17-, K70.4, K71.1, K72-, K76.7, I28.0, R18-, J94.8, K65-, HPHB003, HPJB001, EHCA003, EHCA006, EHCA009, EHCA007, EHCA004, EHCA002, EHCA005, EHCA010, EHCA001, HEPA005, HEPA004, HEPA007
**Dementia**	F00-, F01-, F02-, F03-, F05.1, G30-, G31.1
**Diabetes Mellitus Complicated**	E10.2, E10.3, E10.4, E10.5, E10.7, E11.2, E11.3, E11.4, E11.5, E11.7, E12.2, E12.3, E12.4, E12.5, E12.7, E13.2, E13.3, E13.4, E13.5, E13.7, E14.2, E14.3, E14.4, E14.5, E14.7, H36.0, N08.3, H28.0, G63.2
**Diabetes Mellitus Uncomplicated**	E10.0, E10.1, E10.6, E10.8, E10.9, E11.0, E11.1, E11.6, E11.8, E11.9, E12.0, E12.1, E12.6, E12.8, E12.9, E13.0, E13.1, E13.6, E13.8, E13.9, E14.0, E14.1, E14.6, E14.8, E14.9
**Diabetes Mellitus**	E1
**Disseminated Intravascular Coagulation**	D65-, D68.4
**Gastro Esophageal Varices Bleeding**	I85.0, I98.3, I98.2, EHBD001, EHNE002, HESE001, HESE002
**Gastro Esophageal Varices not Bleeding**	I85.9, I86.4, I98.2, I98.2, EHNE001
**Hemiplegia**	G04.1, G11.4, G80.1, G80.2, G81-, G82-, G83.0, G83.1, G83.2, G83.3, G83.4, G83.9
**Hepatic Encephalopathy**	K72.0, G94.3, R40.2
**Hepatocellular Carcinoma Treatment**	YYYY170, EDLF017, EDLF016, HLNM001, EDQH006, EDLF014, YYYY210, HLHJ005, EDQH007, HLHJ006, EDLF015, EDQH008, HLNK001, EDSF006, EDLL001, ZZQA002, EHSF001, HLQX004, EDLL002, HLFA020

**Hepatocellular Carcinoma**	C22.0
**Hepatopulmonary Syndrome**	I28.0
**Hepatorenal Syndrome**	K76.7
**HIV Infection**	Z21-, B20-, B21-, B22-, B23-, B24-
**Hydrothorax**	J94.8
**Hypercholesterolemia Pure**	E87.0
**Hyperlipemia Mixed**	E78.2
**Hyperlipemia**	E78.0, E78.1, E78.2, E78.4, E78.5
**Hypertension**	I10-, I11-, I12-, I13-, R03.0
**Hypertensive Heart Failure with or without Renal Failure**	I10-, I13.0, I13.2
**Hypertriglyceridemia**	E87.1
**Intrahepatic Bile Duct Carcinoma**	C22.1
**Jaundice**	R17-
**Leukemia**	C91-, C92-, C93-, C94-, C95-, C96-
**Liver Biopsy**	HLHB001, HLHJ003, HLHJ006, HLHH006, HLHJ005, HLHH007, HLHH001, HLHH005
**Liver Disease Mild**	K71.3, K71.4, K71.5, K73-, K74.0, K74.1, K74.2, K74.3, K76.0, K76.4, K76.5
**Liver Disease Moderate to Severe**	K70.3, I85.0, I85.9, I86.4, I98.2, J94.8, K70.3, K70.4, K71.1, K71.7, K72.1, K72.9, K74.4, K74.5, K74.6, K65-, K66-, K76.7, R17-, R18-, EHBD001, EHNE002, HESE001, HESE002, HPHB003, HPJB001, EHCA003, EHCA006, EHCA009, EHCA007, EHCA004, EHCA002, EHCA005, EHCA010, EHCA001, HEPA005, HEPA004, HEPA007
**Liver Transplantation**	HLEA001, HGEA002, HLEA002, HGEA004
**Lung Transplantation**	GFEA004, GFEA001, GFEA006, GGBA001
**Lymphoma**	C81-, C82-, C83-, C84-, C85-, C86-, C87-, C89-, C90-
**Myocardial Infarction**	I21-, I22-, I25.2
**Obesity**	E66-
**Other Cause of Chronic Liver Disease**	E83.0, E83.1, FEJF003, I82.0, K83.0, K74.3, K75.4, Q44.2, Q44.3, Q44.6
**Other Primary Liver Cancer**	C22.2, C22.3, C22.4, C22.7, C22.9
**Palliative Care**	Z51.5
**Pancreatic Steatorrhoea**	K90-
**Pancreatitis Chronic Other**	K86.1
**Paraplegia Hemiplegia**	G04.1, G11.4, G80.1, G80.2, G81-, G82-, G83.0, G83.1, G83.2, G83.3, G83.4, G83.9
**Peptic Ulcer Disease**	K25-, K26-, K27-, K28-
**Peritonitis**	K65-
**Peripheral Vascular Disease**	I70-, I71-, I73.1, I73.8, I73.9, I77.1, I79.0, I79.2, K55.1, K55.8, K55.9, R02-, Z95.8, Z95.9
**Phlebitis and Thrombophlebitis**	I80-
**Pneumocystosis**	B59-

**Pneumonia Bacterial**	J13-, J14-, J15-, J16-, J17.0, J17.8, J18-, J85-
**Pneumonia Due to Streptococcus Pneumoniae**	J13-
**Pneumonia Due to Haemophilus Influenzae**	J14-
**Pneumonia Bacterial not Elsewhere Classified**	J15-
**Pneumonia Due to Other Infectious Organisms not Elsewhere Classified**	J16-
**Pneumonia in Diseases Classified Elsewhere**	J17.0, J17.8
**Portal Hypertension**	K76.6
**Portal Vein Thrombosis**	I81-
**Renal Transplantation**	T86.1, Z94.0, JAEA003
**Smoking**	F17-, Z71.6, Z72.0, T65.2
**Solid Tumor Localized without Liver**	C0, C1, C20-, C21-, C23-, C25-, C26-, C3, C40-, C41-, C43-, C45-, C46-, C47-, C48-, C49-, C5, C6, C70-, C71-, C72-, C73-, C74-, C75-, C76-
**Solid Tumor Localized**	C0, C1, C20-, C21-, C22-, C23-, C24-, C25-, C26-, C3, C40-, C41-, C43-, C45-, C46-, C47-, C48-, C49-, C5, C6, C70-, C71-, C72-, C73-, C74-, C75-, C76-
**Solid Tumor Metastatic**	C77-, C78-, C79-, C80-
**Splanchnic Thrombophlebitis**	I81-, I82-
**Surgical Shunt**	EHCA003, EHCA006, EHCA009, EHCA007, EHCA004, EHCA002, EHCA005, EHCA010, EHCA001, HEPA005, HEPA004, HEPA007
**Thrombophlebitis Migrans**	I82.1
**Transfusion**	FELF003, FELF004
**Transjugular Intrahepatic Posto Systemic Shunt**	EHCF002, EHAF004, EHPF001, EHNF001
**Transjugular Liver Biopsy**	HLHH001, HLHH005
**Transplant Recipient without Liver**	Z94.0, Z94.1, Z94.2, Z94.3, Z94.8, Z94.8, T86.1, T86.2, T86.3, T86.8, T86.8, T86.8, T86.8, Z94801, Z94802, Z94803, Z94804, Z94809, T86.0, JAEA003
**Type 1 Diabetes Mellitus**	E10-
**Type 2 Diabetes Mellitus**	E11-

**Table S2. Age at CFLD Progression and Competing Risks**				
**Event**	Exposure Era	Number of Events (%)	Median (IQR) Age at Event (Years)	*p* value
CFLD progression	**Pre-ETI**	**163 (6.6%)**	**26 (18 - 37)**	
	**Post-ETI**	**63 (0.8%)**	**28 (21 - 39.5)**	**0.177**
Gastroesophageal varices (non-bleeding)	**Pre-ETI**	**75 (3%)**	**20 (15 - 29)**	
	**Post-ETI**	**91 (1.2%)**	**19 (16 - 25.5)**	**0.599**
Gastroesophageal varices bleeding	**Pre-ETI**	**55 (2.2%)**	**25 (17 - 36.5)**	
	**Post-ETI**	**22 (0.3%)**	**24 (20 - 37)**	**0.604**
Decompensated cirrhosis	**Pre-ETI**	**126 (5.1%)**	**26 (19 - 37)**	
	**Post-ETI**	**46 (0.6%)**	**26.5 (20 - 36.8)**	**0.634**
Acute liver failure	**Pre-ETI**	**90 (3.6%)**	**29 (22 - 40)**	
	**Post-ETI**	**56 (0.7%)**	**27.5 (21.8 - 37)**	**0.537**
Hepatocellular carcinoma	**Pre-ETI**	**9 (0.4%)**	**56 (41 - 62)**	
	**Post-ETI**	**3 (0%)**	**58 (54 - 67)**	**0.46**
Liver transplantation	**Pre-ETI**	**26 (1.1%)**	**22.5 (18 - 29.5)**	
	**Post-ETI**	**2 (0%)**	**23 (22 - 24)**	**1**
Lung transplantation	**Pre-ETI**	**509 (20.6%)**	**28 (23 - 36)**	
	**Post-ETI**	**84 (1.1%)**	**30 (21 - 39)**	**0.856**
Death without transplantation	**Pre-ETI**	**242 (9.8%)**	**40 (27 - 65)**	
	**Post-ETI**	**185 (2.4%)**	**51 (36 - 73)**	**< 0.001**

This comparison captures temporal associations and does not reflect individual-level exposure to ETI. The ETI era commenced in France after December 2019. *p* values were computed using Wilcoxon Rank-Sum Test. Abbreviation: CFLD = cystic fibrosis liver disease; ETI = Elexacaftor, Tezacaftor, and Ivacaftor; IQR = interquartile range

Table S3. Characteristics of pwCF, by CFLD Progression

Characteristic	Overall N = 3,814[1]	No CFLD Progression n = 3,697, 96.9%[1]	CFLD Progression n = 117, 3.07%[1]	*p* value[2]
**Censored during the ETI era**	1,907 (50.00%)	1,875 (50.72%)	32 (27.35%)	<0.001
**Age at censoring**	20 (18, 26)	20 (18, 25)	26 (19, 39)	<0.001
**Male sex**	2,021 (52.99%)	1,949 (52.72%)	72 (61.54%)	0.060
**Smoking habits**	190 (4.98%)	171 (4.63%)	19 (16.24%)	<0.001
**Alcohol use disorders**	89 (2.33%)	61 (1.65%)	28 (23.93%)	<0.001
**Liver risk factors**	52 (1.36%)	34 (0.92%)	18 (15.38%)	<0.001
**Type-2 diabetes mellitus**	799 (20.95%)	733 (19.83%)	66 (56.41%)	<0.001
**Obesity**	135 (3.54%)	123 (3.33%)	12 (10.26%)	<0.001
**HIV-infection**	7 (0.18%)	4 (0.11%)	3 (2.56%)	<0.001
**CCI ≥ 3**	415 (10.88%)	386 (10.44%)	29 (24.79%)	<0.001
**Deprivation quintiles 4 or 5**	1,485 (38.94%)	1,441 (38.98%)	44 (37.61%)	0.8
**Death without transplantation**	234 (6.14%)	207 (5.60%)	27 (23.08%)	<0.001
**Follow-up duration (months)**	34 (17, 54)	34 (17, 54)	36 (23, 54)	0.2

[1] n (%); Median (Q1, Q3)

[2] Pearson’s Chi-squared test; Wilcoxon rank sum test; Fisher’s exact test

CFLD progression was any of decompensated cirrhosis, portal hypertension bleeding, primary liver cancer, liver transplantation, or lung transplantation without prior CFLD progression. The Charlson Comorbidity Index was categorized into low (<3), intermediate (3–5), and high (>5) scores to reflect increasing levels of frailty. The French Deprivation Index was used to assess spatial socioeconomic and health inequalities, with scores divided into quintiles. The ETI era commenced in France after December 2019. Abbreviations: CCI = Charlson Comorbidity Index; CFLD = cystic fibrosis liver disease; ETI = Elexacaftor-Tezacaftor-Ivacaftor; FDEP = French Deprivation Index; pwCF = people with cystic fibrosis.

Fig. S1. Cumulative Incidence of CFLD Progression in pwCF Aged ≥12 Years by ETI era

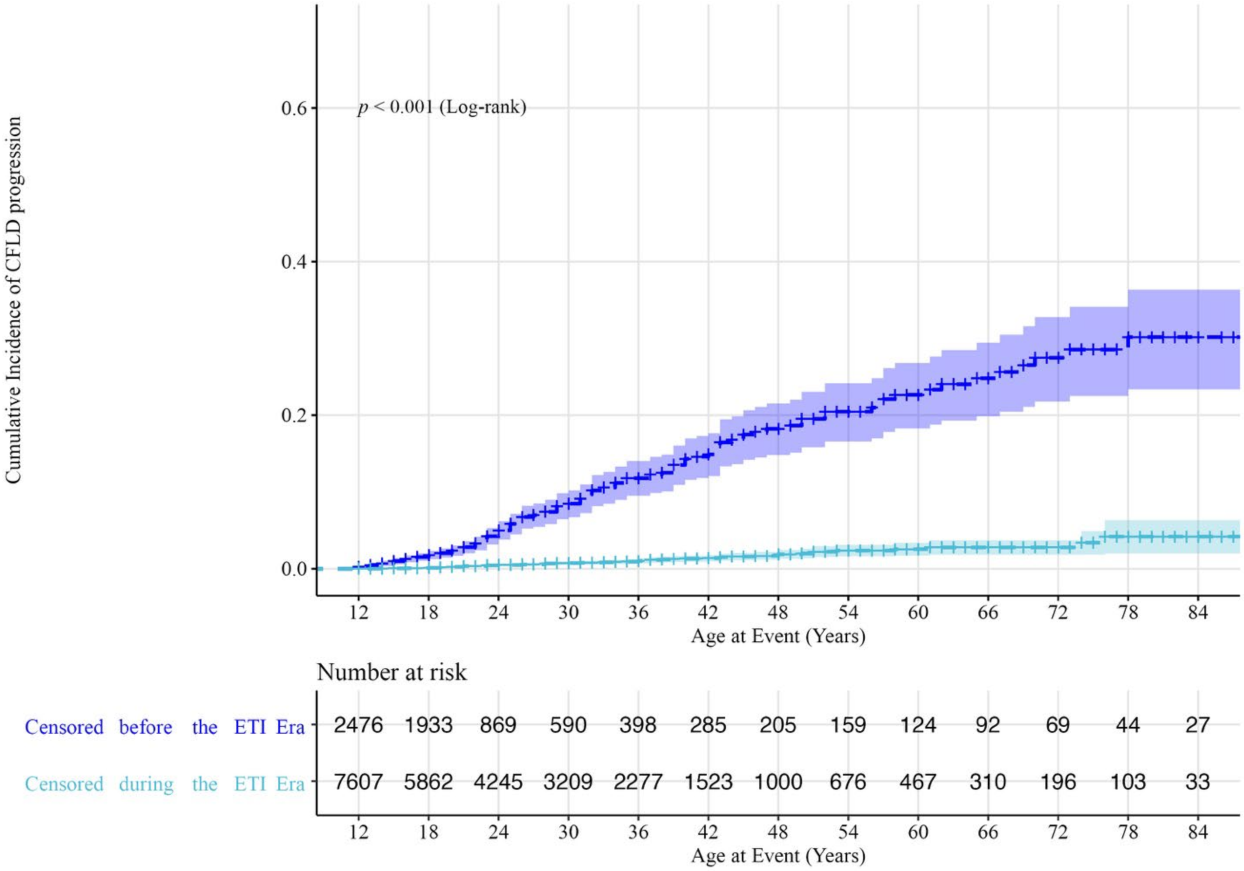

Kaplan–Meier estimates of CFLD progression probability in pwCF aged 12 years and older, stratified by whether they were censored before or after the introduction of ETI on December 15, 2019. This comparison reflects temporal trends but does not represent individual-level exposure to ETI. The total follow-up time was 75,276 person-months (6,273 person-years) in the pre-ETI era and 640,659 person-months in the post-ETI era (53,388 person-years). Abbreviations: CFLD = cystic fibrosis liver disease; ETI = Elexacaftor, Tezacaftor, and Ivacaftor, pwCF =people with cystic fibrosis.

Fig. S2. Covariate Balance Before and after Weighting in the Propensity Score–matched Sensitivity Analysis Cohort

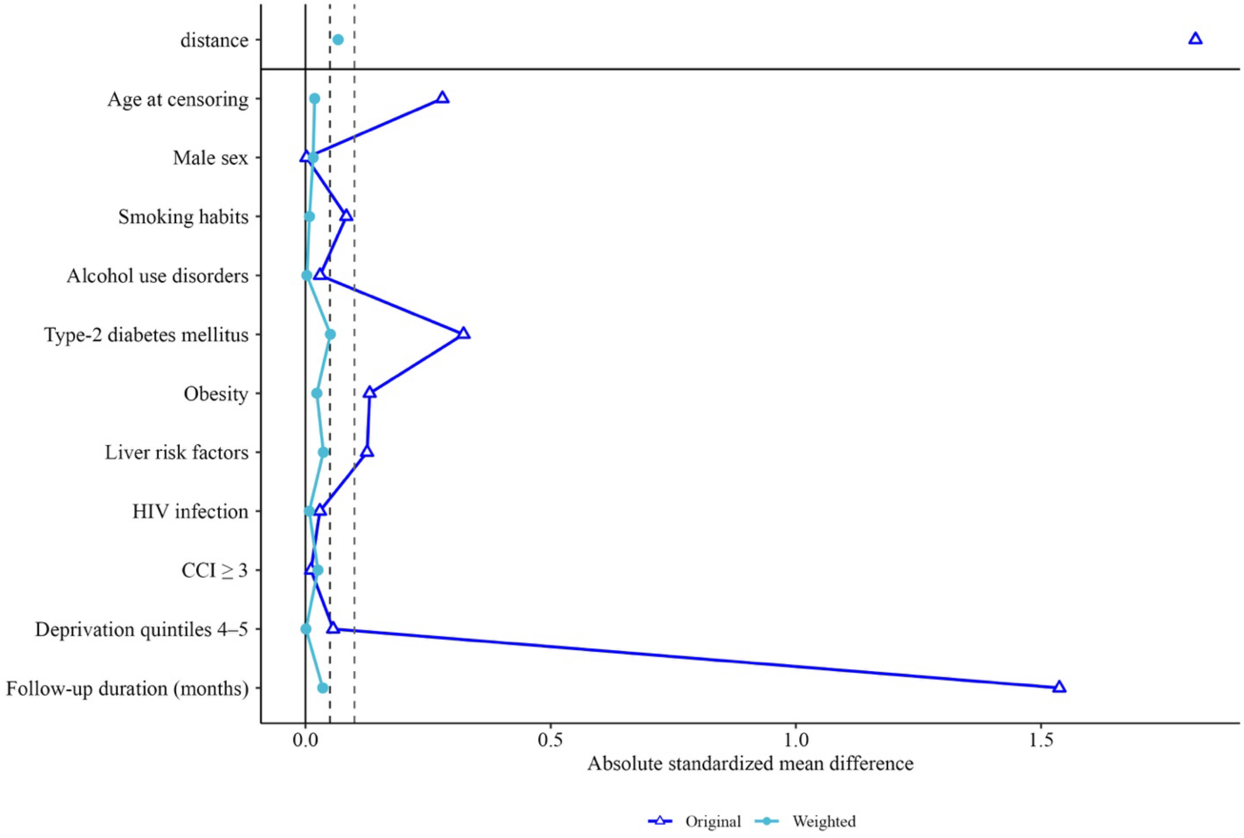

Propensity scores were estimated using logistic regression and matched by ETI period using a nearest-neighbor algorithm with a caliper of 0.1. Standardized mean differences for baseline characteristics before (unweighted) and after (weighted) propensity score matching among pwCF included in the analytic cohort. A threshold of 0.1 (dashed vertical line) denotes acceptable balance. Abbreviations: CCI = Charlson Comorbidity Index
